# Soft chromophore featured liquid porphyrins and their utilization toward liquid electret applications

**DOI:** 10.1038/s41467-019-12249-8

**Published:** 2019-09-30

**Authors:** Avijit Ghosh, Manabu Yoshida, Kouji Suemori, Hiroaki Isago, Nagao Kobayashi, Yasuhisa Mizutani, Yuki Kurashige, Izuru Kawamura, Masami Nirei, Osamu Yamamuro, Tomohisa Takaya, Koichi Iwata, Akinori Saeki, Kazuhiko Nagura, Shinsuke Ishihara, Takashi Nakanishi

**Affiliations:** 1 International Center for Materials Nanoarchitectonics (WPI-MANA), National Institute for Materials Science (NIMS), 1-1 Namiki, Tsukuba, 305-0044 Japan; 2 Flexible Electronics Research Center, National Institute of Advanced Industrial Science and Technology (AIST), 1-1-1 Umezono, Tsukuba, 305-8565 Japan; 3 Research Center for Functional Materials (RCFM), National Institute for Materials Science (NIMS), 1-2-1 Sengen, Tsukuba, 305-0047 Japan; 4 Smart Material Science and Technology, Interdisciplinary Graduate School of Science and Technology, Shinshu University, 3-15-1 Tokida, Ueda, 386-8567 Japan; 5 Department of Chemistry, Graduate School of Science, Osaka University, 1-1 Machikaneyama, Toyonaka, Osaka 560-0043 Japan; 6 Department of Chemistry, Graduate School of Science, Kyoto University, Oiwake-Cho, Kitashirakawa, Sakyo, Kyoto, 606-8502 Japan; 7 Department of Materials Science and Engineering, Graduate School of Engineering, Yokohama National University, 79-5 Tokiwadai, Yokohama, Kanagawa 240-8501 Japan; 8 The Institute for Solid State Physics, The University of Tokyo, 5-1-5 Kashiwanoha, Kashiwa, Chiba 277-8581 Japan; 9 Department of Chemistry, Faculty of Science, Gakushuin University, 1-5-1 Mejiro, Toshima, Tokyo, 171-8588 Japan; 10 Department of Applied Chemistry, Graduate School of Engineering, Osaka University, 2-1 Yamadaoka, Suita, Osaka 565-0871 Japan

**Keywords:** Organic molecules in materials science, Self-assembly, Devices for energy harvesting, Fluids

## Abstract

Optoelectronically active viscous liquids are ideal for fabricating foldable/stretchable electronics owing to their excellent deformability and predictable π-unit–based optoelectronic functions, which are independent of the device shape and geometry. Here we show, unprecedented ‘liquid electret’ devices that exhibit mechanoelectrical and electroacoustic functions, as well as stretchability, have been prepared using solvent-free liquid porphyrins. The fluidic nature of the free-base alkylated-tetraphenylporphyrins was controlled by attaching flexible and bulky branched alkyl chains at different positions. Furthermore, a subtle porphyrin ring distortion that originated from the bulkiness of alkyl chains was observed. Its consequences on the electronic perturbation of the porphyrin-unit were precisely elucidated by spectroscopic techniques and theoretical modelling. This molecular design allows shielding of the porphyrin unit by insulating alkyl chains, which facilitates its corona-charged state for a long period under ambient conditions.

## Abstract (web summary)

Though electret materials are attractive for realizing flexible mechanoelectrical devices, these materials are typically solid films. Here, the authors report stretchable ‘liquid-electret’ devices consisting solvent-free liquid porphyrins that show piezoelectric and electroacoustic functionality.

## Introduction

Flexible mechanoelectrical devices have generated broad interest for energy production in self-powered wearable electronics that include human healthcare sensors, surgery tools, muscle-driven energy scavengers, communication devices, and smart textiles^1,2^. Electret materials^3,4^, possessing a quasi-permanent electric charge or dipole polarisation (ferroelectric) that can be converted to electrical energy by mechanical force, are one of the key components for powering such mechanoelectrical devices. Conventional electret materials used in such devices are generally solid films composed of insulating polymeric materials^5,6^. Solid materials possessing intrinsic dipoles, high dielectric properties and high surface charge densities are generally known to exhibit promising electret properties. Several factors for the molecular design of electret are considered including the surface polarity, conjugation length, hydrophobicity, morphology, architecture, donor/acceptor strength, and interfacial energy barrier, which can affect charge storage properties^7^. The existence of liquid-electrets, in which the liquids have a net electrostatic charge, e.g. charged droplets of liquid analyte produced in the electrospray mass spectrometer^8^, charged liquid micelles^9^, and liquids containing charged colloidal particles^10^, is possible in principle. However, the charge storage potentiality of liquid materials has never been considered a power source in electret devices that could advance the technical applications of stretchable/wearable mobile electronics^11^, human patches^12^, and electrode-less electrogeneses^13^. In fact, the fundamental properties of a liquid (e.g. fluidity with fast diffusion of molecules, easy processing, defect-free, and ultimate flexibility with high deformability) are prerequisites for the promotion of flexible/stretchable device technologies. Herein, the design concept of solvent-free molecular liquid-electrets is presented, which are non-volatile substances capable of holding a (pseudo)-permanent charge. The present strategy of creating a liquid-electret material involves shielding of a π-conjugate molecular unit with insulating and flexible bulky-alkyl chains^14^, wherein porphyrin was chosen as the charge-storing π-unit. The hydrophobic and insulating nature of the alkyl chains stabilised the trapped charge rendering from air and electrostatic interactions. Moreover, the fluidic nature of the liquid-electret was beneficial for providing maximum device deformability even in the bent and stretched states.

In this report, first the molecular design strategies of liquid porphyrins with a shielded π-unit have been discussed, followed by a guide to fine-tuning of their liquid-physical properties by altering the substitution positions of the branched alkyl chains on the periphery of tetraphenylporphyrin. Because of attaching highly bulky and flexible alkyl chain moieties, a subtle porphyrin ring distortion was observed. The impact of this distortion on the electronic perturbations have been investigated in detail. The non-planar conformational changes of the π-units^15^, in particular the π-extended macrocycles^16–18^ and many biological systems^19^, are crucial for controlling their optoelectronic functions. Finally, as a proof-of-concept, the shielded π-units of porphyrin and their inherent liquid nature were considered together and revealed unconventional liquid-electret-based flexible and stretchable mechanoelectrical devices, which are of practical importance for the development of wearable electret^20^ and nanogenerator^21^ applications.

## Results

### Molecular design and characterisation

**Fig. 1:**
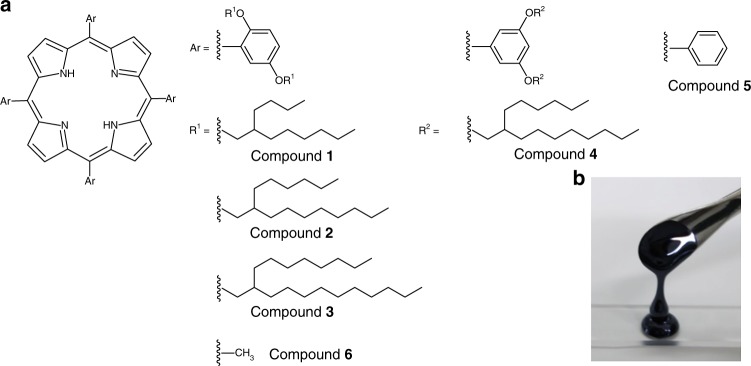
Molecular design of liquid porphyrins. **a** Chemical structures of compounds **1**–**6**. The branched alkyl chains of different chain lengths such as 2-butyloctyl (compound **1**), 2-hexyldecyl (compounds **2** and **4**) and 2-octyldodecyl (compound **3**) were attached on the *meso*-phenyl groups of tetraphenylporphyrin (compound **5**) through an ether linkage. In compounds **1**–**3**, the alkyl chains were attached at the 2,5-positions (these positions are considered with respect to the phenyl carbon which is attached to the *meso*-carbon of the porphyrin ring), while in compound **4**, the attachment was at the 3,5-positions of the *meso*-phenyl groups. Compound **5** was used as a standard and compound **6** was considered as a model compound for theoretical simulation. **b** Photo image of the solvent-free liquid-physical appearance of compound **4** at 296 K

The porphyrin derivatives (compounds **1**–**4**, Fig. 1a) were designed by attaching Guerbet alcohol-based^22^ branched alkyl chains on the *meso*-phenyl groups of 5,10,15,20-tetraphenylporphyrin (H_2_TPP, compound **5**). The compounds were successfully synthesised (Supplementary Fig. 1) and unambiguously characterised by nuclear magnetic resonance (NMR) spectroscopy (Supplementary Figs. 2–9) and matrix-assisted laser desorption ionisation-time of flight (MALDI-TOF) mass spectrometry (Supplementary Figs. 10–13). The liquid porphyrins were dried under high vacuum at 323 K overnight to ensure the absence of any trace amount of trapped solvent. It is known that the alkyl substituents at the 2-position of the phenyl unit are important for isolating the π-core of liquid pyrenes^23^ and reducing the viscosity of liquid fullerenes^24^. The compounds **2** and **4** were designed to reveal the effect of alkyl substituents at the 2-position of *meso*-phenyls on the shielding of  the porphyrin π-core as well as their influence on the optical, thermal, and rheological properties in the liquid-state.

### Liquid physical properties

**Fig. 2:**
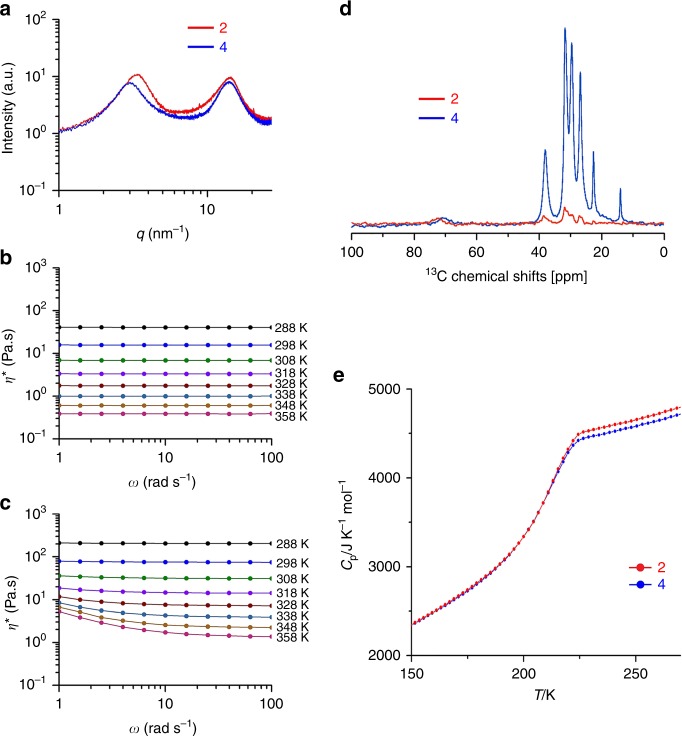
Impact of the positions of alkyl substituents on the liquid physical properties. **a**, SWAXS profiles of compounds **2** and **4** at 296 K. **b**, **c** Change of complex viscosity (η*) of compounds **2** (**b**) and **4** (**c**) as a function of angular frequency *ω* at various temperatures ranging from 288 to 358 K. **d** Comparison of magic-angle spinning (MAS) ^13^C NMR spectra of compounds **2** and **4** in cross-polarisation (CP)-MAS mode, which was collected in the solvent-free liquid-state under MAS of 12.5 kHz at 296 K. **e** Heat capacity (*C*_*p*_) measurements by using a custom-made calorimeter under adiabatic condition

Compounds **1**–**4** were obtained as viscous fluids at 296 K in solvent-free condition. In polarised optical microscopy (POM), no birefringence texture appeared for these fluids at ambient temperature and even after storing at 277 K for several months, indicating their amorphous state and the absence of long-range ordered structures (Supplementary Fig. 14). The photoconductivity maximum (*ϕ*Σ*μ*_max_) values of ~1.0 × 10^−5^ cm^2^ V^−1^ s^−1^, obtained for compounds **2** and **4** by the flash-photolysis time-resolved microwave conductivity (FP-TRMC)^25^ technique (Supplementary Fig. 15), further confirmed the lack of long-range ordering of porphyrin units. These *ϕ*Σ*μ*_max_ values are one order of magnitude smaller than those of well-ordered porphyrin assemblies measured by the same technique^26^. The amorphous nature of these liquids was also investigated by small- and wide-angle X-ray scattering (SWAXS) analysis (Fig. 2a). Both compounds **2** and **4** exhibited two broad halos, which are typically observed for alkylated-π liquid systems^23,27^. The halo at lower *q* region was assigned to the distributed distance between the randomly oriented π-rich TPP units that were separated by bulky alky side-chains. The average TPP-TPP separation distance in compound **2** (18.5 Å) was shorter than that in compound **4** (20.9 Å). This is because of the alkyl chains at 3,5-positions, which are distributed outward from the TPP-unit of compound **4** (density (*d*) = 0.97 g/cm^3^) and make its effective volume larger than compound **2** (*d* = 0.98 g/cm^3^), where the alkyl chains at 2*-*position can flip inward over the porphyrin-unit.

The higher loss moduli (*G*″) over storage moduli (*G*′) obtained in the rheological measurements confirmed the liquid characteristics of compounds **1**–**4** at 298 K (Fig. 2b, c and Supplementary Fig. 16). Importantly, compound **2** behaved as a Newtonian-type fluid while compound **4** acted as a non-Newtonian-type, which were evidenced by their angular frequency (*ω*), and independent and dependent complex viscosity (η*), respectively. At relatively high temperatures, the decrease of η* with increasing *ω* (Fig. 2c) and strain amplitude dependent modulus behaviour (Supplementary Fig. 16c) indicated a shear-thinning behaviour of compound **4**. In addition, compound **4** exhibited η* that was five-fold that of compound **2**.

In order to investigate the fluidity on the molecular motion correlation time scale, the magic-angle spinning (MAS) solid-state NMR (SSNMR) technique was applied to neat samples. Although the proton signals were broad, the features of ^1^H NMR spectra of compounds **2** and **4** in this neat state (Supplementary Fig. 17) resembled those in solution (Supplementary Figs. 4 and 8). Compared to compound **4**, relatively sharper and better-resolved signals appeared for compound **2**. In cross-polarisation MAS (CP-MAS) mode, static ^1^H–^13^C dipolar interactions increase CP efficiency and hence the intensity of ^13^C NMR signals increase in the correlation time scale of 10^−2^–10^−4^ s^28^. Experimentally in CP-MAS mode (Fig. 2d and Supplementary Fig. 18a), ^13^C NMR signals of the alkyl carbons of compound **4** appeared as sharper and more intense signals than those of compound **2**. While in direct single pulse excitation with the proton-decoupling MAS (DD-MAS) mode, ^13^C NMR experiments with short spin-lattice relaxation time (*T*_1_) afforded increased signal intensity due to decreased signal saturation. ^13^C (*T*_1_) value decreases significantly in the correlation time scale of 10^−5^–10^−8^ s^28^. In DD-MAS mode, the alkyl carbon signals of compound **2** were relatively sharp and intense (Supplementary Fig. 18b). These results revealed a faster molecular motion of compound **2** over compound **4** in terms of alkyl chain mobility. This faster molecular motion could also be correlated to the lower liquid bulk-viscosity according to the Stokes-Einstein relation (*τc* = 4*πηa*^3^/*kT*, where *τc*, *η*, *a*, *k*, and *T* are correlation time, viscosity, molecular radius, Boltzmann’s constant and absolute temperature, respectively) observed for compound **2** than that of compound **4** as discussed before.

To gain further insight into the liquid characteristics and differences in the fluidity of compounds **2** and **4**, their heat capacities were measured by using a custom-made adiabatic calorimeter^29^. A heat capacity jump (*∆C*_*p*_) accompanying enthalpy relaxation, which is characteristic of glass transition, appeared at ~208 K for both compounds **2** and **4** (Fig. 2e). The magnitude of *∆C*_*p*_ (~1000 J K^−1^ mol^−1^) was much larger than those of common glass forming liquids; e.g. the *∆C*_*p*_ of a typical glass-former orthoterphenyl (OTP) is 110 J K^−1^ mol^−1^^30^. The large *∆C*_*p*_ could be attributed to the tremendous conformational disorder of the long and branched alkyl chains of compounds **2** and **4**. It is also interesting that *∆C*_*p*_ in the case of compound **2** was 2% larger than that of compound **4** at temperatures above *T*_g_. This possibly reflects the low viscosity and less ordered local structure of compound **2**. The stable thermal properties^17,31,32^ of compounds **2** and **4** were also confirmed by differential scanning calorimetry (DSC) and thermogravimetric analysis (TGA) (Supplementary Figs. 19 and 20).

### Optical properties

**Fig. 3:**
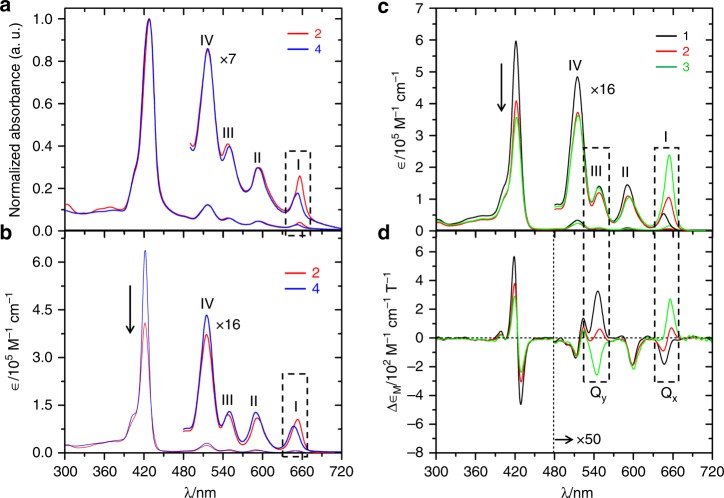
Electronic properties of porphyrin unit. **a**, **b** Comparison of UV-Visible absorption spectra of compounds **2** and **4** in the solvent-free liquid state (normalised at Soret-band peak intensity) (**a**) and dichloromethane solution (**b**). The four Q-bands are designated as **I**–**IV**. **c**, **d**, UV-Visible absorption (**c**) and the corresponding MCD spectra (**d**) of compounds **1–3** recorded in dichloromethane. The systematic changes in the Q-bands are shown at bands **I** and **III**. Solutions having concentrations 1.2 × 10^−6^ M and 2 × 10^−5^ M were used for recording the Soret and Q-band spectra, respectively, in dichloromethane

The UV-Visible absorption spectra of solvent-free liquids compounds **2** and **4** exhibited one strong Soret band at 427–428 nm and four weak Q-bands (marked as **I**–**IV**, Fig. 3a and Supplementary Table 1) in the range of 514–654 nm. A similar spectral feature was also observed in their dichloromethane solution (Fig. 3b), indicating sufficient shielding of the porphyrin-unit by the alkyl side-chains. However, both the Soret and Q-bands in the solvent-free state were slightly broadened and red shifted by 5–7 nm, which could be attributed to the close but random association of molecules^33^.

Unexpectedly, a slight red-shift and subtle enhancement in the intensity of the lowest energy Q-band (**I**) of compound **2** was observed as compared to that of compound **4**, which was only visible after many fold magnifications of its intensity (insets of Fig. 3a, b). In addition, the Soret band in the solution spectrum of compound **2** was of lower intensity than that observed in the spectrum of compound **4** (Fig. 3b). More importantly, the intensity order of the Q-bands of compound **2** did not follow the order **IV** > **III** > **II** > **I**, while those of compound **4** did, which is normally observed in the standard molecule **5** (Supplementary Fig. 21). These subtle differences in the absorption spectra may hint at the non-identical electronic structures of compounds **2** and **4**. Furthermore, the alkyl protons of compound **2** observed in solution ^1^H NMR were found to be shifted slightly more upfield than compound **4** (Supplementary Fig. 22), revealing the close association of the porphyrin unit and the 2-position alkyl chains in compound **2** that were influenced by the macrocyclic ring current. Thus, the electronic perturbation in compound **2** appeared to be affected by the bulky-alkyl side-chains at the 2-position, which restricted the rotation of the *meso*-phenyl groups. Therefore, the fundamental impact of the bulkiness of the alkyl side chains at the 2,5-positions on the electronic perturbation of porphyrin-unit was further studied using a series of derivatives compounds **1**–**3** (Fig. 1a) having different alkyl chain lengths. For instance, a gradual enhancement in the bulkiness was presumed with the increasing lengths of the alkyl side chains from compounds **1** to **3** and the following experiments were therefore performed to test this hypothesis.

### Effect of alkyl chain on electronic perturbation

The UV-Visible absorption spectral features in the dichloromethane solutions of compounds **1**–**3** were essentially similar except for a few small differences in the Q-band region (Fig. 3c). With increasing alkyl chain lengths, band-**I** was slightly red shifted with a concomitant increase in intensity. Interestingly, compounds **2** and **3** did not follow the Q-bands intensity order **IV** **>** **III** **>** **II** **>** **I**. Gouterman’s “Four-Orbital” model^34,35^ attributes this anomalous behaviour in intensity of the absorption spectra to the energy gaps between the two highest occupied molecular orbitals (HOMO and HOMO‒1; i.e. *∆*HOMO) and two lowest unoccupied molecular orbitals (LUMO and LUMO + 1; i.e. *∆*LUMO) (for details, see Supplementary Fig. 23).

Here, the relative magnitudes of *∆*HOMO and *∆*LUMO were investigated by applying magnetic circular dichroism (MCD) spectroscopy for a fundamental understanding of the ground and excited state degeneracy arising from the Zeeman splitting of the electronic states^36–38^. The negligibly small changes in the UV-Visible absorption spectra, particularly at Q-bands **I** and **III** (Q_*x*_ and Q_*y*_, respectively), were found to be significant and more prominent in their corresponding MCD spectra (Fig. 3d, for details, see Supplementary Note 1). The signs of Q_*x*_ and Q_*y*_ gradually became opposite with increasing chain lengths in compounds **1**–**3**. This unique reversal of the MCD-sign sequence is rarely observed for free-base porphyrinoids^39–41^. On considering Michl’s^42^ “soft MCD chromophores” model, it was inferred that the gradual increase in the relative value of *∆*LUMO with increasing chain lengths was responsible for such reversal of the MCD-sign sequences (Supplementary Fig. 24). It should be noted that an identical trend of MCD-sign sequences was also observed in their solvent-free liquid-state (Supplementary Fig. 25).

This kind of perturbation in *∆*LUMO is mainly known to occur either by introduction of electron-withdrawing/donating substituents at the periphery of porphyrin-unit, which can directly influence the π–system, as observed for chlorins or bacteriochlorins^39–41^ or because of the macrocyclic ring distortion. However, in the case of compounds **1**–**3**, the first explanation can be disregarded because only the length of the alkyl chain is changed (increases). Therefore, structural distortion may be the most probable reason for the reversal of MCD signs^38^. The non-planar distortion of the porphyrin ring in compounds **1**‒**3** was further confirmed by subsequent resonance Raman (RR) measurements^43^ and theoretical model calculations.

### Subtle ring distortion with increasing alkyl chain lengths

**Fig. 4:**
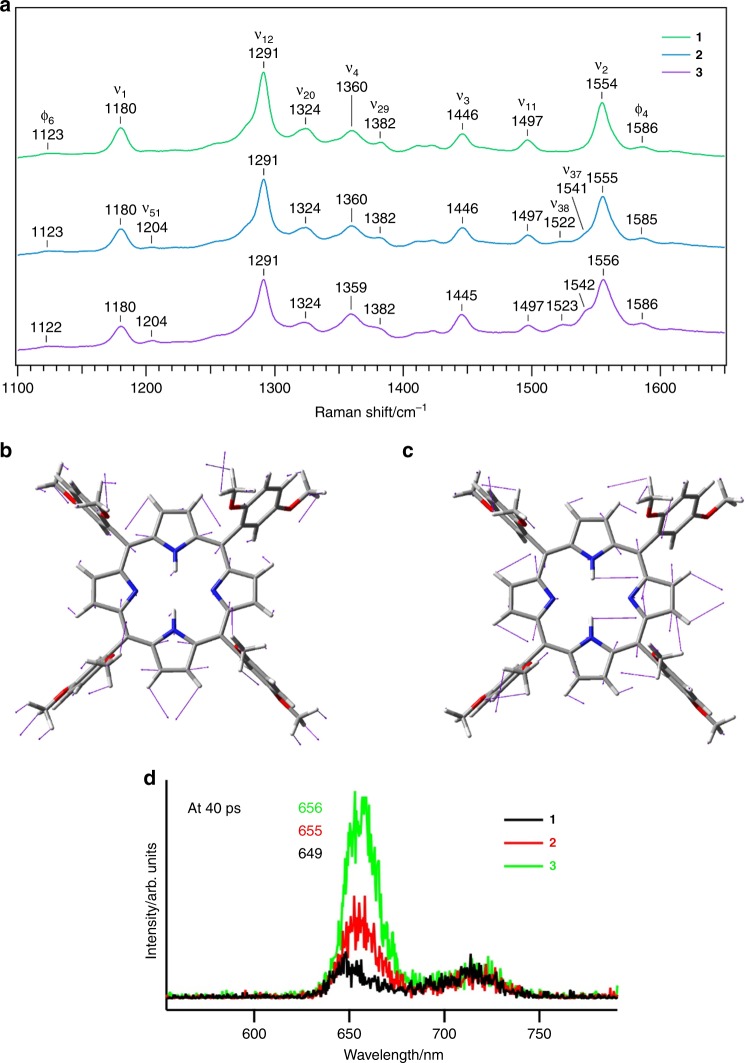
Experimental evidence of subtle porphyrin ring distortion. **a** Resonance Raman spectra for compounds **1**‒**3** in the high frequency region recorded by exciting at 405 nm in dichloromethane at room temperature. With increasing chain lengths, the three bands centred at 1204, 1522–1523, and 1541–1542 cm^–1^ become prominent. **b**, **c** Eigenvectors calculated for the two E_u_ modes ν_37_ at 1535 cm^–1^ (**b**) and ν_38_ at 1545 cm^–1^ (**c**). These modes are associated with out-of-plane stretching of porphyrin ring. **d** Time-resolved fluorescence spectra of compounds **1**‒**3** collected at 40 ps of excitation, measured in toluene by exciting at 400 nm

While the RR spectra of compounds **1**–**3** in dichloromethane (Fig. 4a) were quite similar with few systematic changes in the range of 1100–1650 cm^‒1^, they were nearly identical in the 600–1100 cm^‒1^ region (Supplementary Fig. 26). Mode assignments (Supplementary Note 2) were made based on the comparison of the RR spectrum and the DFT normal mode analysis for free-base H_2_TPP^44^ in *D*_4*h*_ symmetry (vide infra) since the spectra of compounds **1**–**3** were similar to that of H_2_TPP. The new bands at 1522–1523 and 1541–1542 cm^‒1^ become prominent in compounds **2** and **3** with the increasing length of the alkyl chains, while these bands were absent in compound **1** (Fig. 4a). The newly appearing bands were assigned to the E_u_ modes in *D*_4*h*_ symmetry on the basis of frequencies of 1535 and 1545 cm^–1^ (Fig. 4b, c) obtained by the DFT calculations (Supplementary Table 2). The *D*_4*h*_ symmetry of the porphyrin macrocycle was likely lowered in compounds **2** and **3** to either of its subgroups because the E_u_ mode was symmetrical and thus RR inactive. Moreover, the appearance of the E_u_ bands at ~1523 and ~1542 cm^‒1^ indicated the non-planarity of the porphyrin macrocycles in compounds **2** and **3**; this is because symmetry lowering to the *D*_2*h*_ (planar) structure does not result in the increase of Raman or RR active modes (Supplementary Table 3). The bands at 1522–1523 and 1541–1542 cm^‒1^ should therefore be assigned to the E modes (in-plane vibration) of *D*_2*d*_ (saddled) and *S*_4_ (ruffled) symmetries^45^.

Similarly, the band at 1204 cm^‒1^ in compounds **2** and **3** was assigned to another E_u_ vibrational mode ν_51_, which appeared at 1248 cm^‒1^ in the DFT calculations. These findings showed that the porphyrin macrocycle became less planar with the increasing chain length. The changes in the orbital energies (Supplementary Table 4) were calculated by the DFT, in addition to the normal modes characterised by the saddling distortion or ruffling distortion (Supplementary Fig. 27), which lower the molecular point group symmetry from *D*_*4h*_ to *D*_*2d*_ and *S*_*4*_, respectively. The effect of increasing distortions of the porphyrin ring was further reflected in the monotonous decrease in the fluorescence lifetime in the order of compound **1** > compound **2** > compound **3** (Fig. 4d and Supplementary Fig. 28). The non-planarity accelerated the non-radiative decay, which resulted in a decrease in the fluorescence lifetime. The observed spectral changes could mainly be explained by ring distortion, while the length of alkyl chains in the series compounds **1**‒**3** will not perturb their electronic state in a direct manner. The effects of ring distortion on the electronic state of porphyrin are still controversial^46^, because the electronic state could be perturbed by the use of a variety of peripheral substituents and/or central-metal ions in addition to the non-planar ring distortion, as shown in previous studies^47,48^. Therefore, these results provide direct evidence that the porphyrin ring distortion substantially influences its electronic states.

### Liquid electret applications

An electret is an insulating dielectric material that exhibits quasi-permanent charge or dipole moment in the bulk and hundreds of potential voltage on its surface, which can be used to provide a self-sustaining bias voltage for electrostatic power generators^3–6^. In this study, alkyl insulated porphyrin-units were used to illustrate the uncommon liquid-electret properties that allowed holding of the quasi-electric charges on corona-charging under the facile device fabrication process (Methods and Supplementary Fig. 29). It should be noted that both compounds **2** and **4** exhibited similar electret performance; consequently, further discussions are based on the results of liquid **2** as a representative case.

**Fig. 5:**
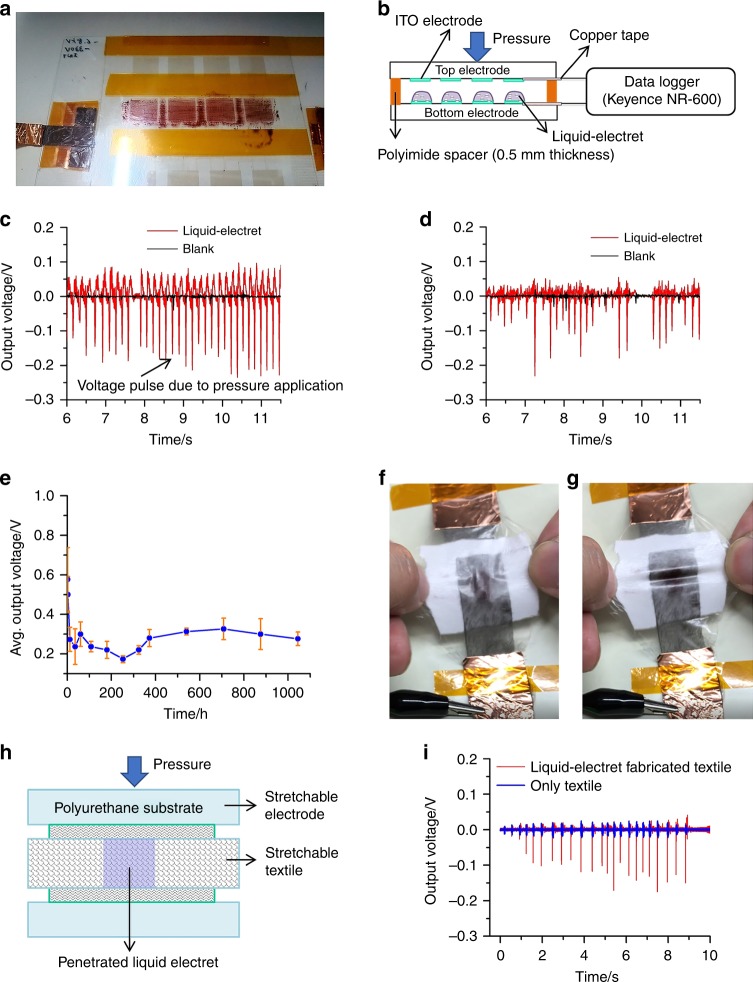
Liquid electret characteristics exhibited by liquid porphyrin. **a** Photograph of the prototype liquid-electret device. **b** Schematic diagram of the cross-sectional view of the constructed liquid-electret device. **c**, **d** Output voltage characteristics of the liquid-electret device in its initial state (**c**) and after 7 days (**d**). **e** Decay graph of liquid-electret device showing the stability of average output voltage (V) on time scale (hour). The error bars represent the standard deviation of three measurements. **f**, **g** Photographs of the stretchable liquid-electret device prepared with a stretchable textile; before stretching (**f**) and after stretching (**g**). **h** Schematic diagram of the cross-sectional view of the stretchable liquid-electret device. **i** Comparison of output voltage characteristics of the stretchable liquid-electret device with textile in the presence and absence of liquid-electret porphyrin

After the completion of corona charging, the surface potential of the liquid compound **2** was measured to be −330 V. Next, a sandwich-type electret device was fabricated (Fig. 5a, b) in which the distance between the electrodes was maintained at 0.5 mm by using a polyimide spacer. When the mechanical stress (pressure) was applied to the upper anode electrode of the device, a voltage pulse of about −0.1 V was generated (Fig. 5c). It was found that even after storing the device for seven days under ambient conditions, the mechanoelectrical effect remained active (Fig. 5d), indicating that the stable electret state could be maintained for prolonged time. The stability of this liquid-electret device was further studied by examining its decay by measuring the average output voltage (V) as a function of time (Fig. 5e) under ambient conditions. The pressure was applied by letting a 10 g metal block fall freely from an exact 10 cm height above the device surface. A fast decay of voltage was observed during the initial 12 h and after that the average output voltage stabilised over the experimental duration of 1044 h (43.5 days).

This liquid-electret device showed similar electret properties compared to conventional solid-electret film. For instance, the standard solid film-type-electret composed of copolymer of tetrafluoroethylene and perfluoroalkoxyethylene (PFA)^49^ exhibited similar intensity of voltage signals as that of the liquid-electret (Supplementary Fig. 30).

In a control experiment, on corona charging to the individual constituent components of liquid porphyrin compound **2**, i.e. analogous solid compound **5** and the pure liquid alkyl chain (*2*-hexyl-*1*-decanol), exhibited negligible static electric surface voltages (~−0.05 V and −0.02 V respectively, after 30 min of corona charging), indicating their non-electrified state (Supplementary Fig. 31). Thus, these experiments suggested that both the liquid feature (which, decreased its semiconducting properties) and suitable functional π-core were complementary to each other; a conclusion that is relevant while designing such unconventional liquid-electret materials.

The most distinguishing characteristic of the liquid-electret is its free shape and deformability, which was further integrated into a prototype stretchable device as demonstrated in Fig. 5f–h (Supplementary Movie 1). Polyurethane and silver-plated nylon fibres were employed as stretchable materials^50^ and because of its free-flowing properties, the liquid porphyrin could directly penetrate a stretchable textile (Fig. 5h). By applying finger-pressure on this stretchable device, voltage pulses of around −0.1 V were observed, while in case of only textile, no such mechanoelectrical effect was observed (Fig. 5i).

The electroacoustic performance of this liquid-electret was further tested by integrating the device with an AC inverter and a vibration sensor (Supplementary Fig. 32). The oscillation characteristics measurement of this device confirmed ~200 Hz oscillation frequency output, which is an audible sound, while applying 1 kHz input frequency from the AC inverter. At this stage, it was assumed, the viscoelastic properties of the liquid-electret could explain the downshift of the output frequency.

The fate of the charged–species (positive/negative ions) in the π-core of the liquid porphyrin units was further examined by absorption spectral analysis. A thin film of compound **2** coated on the conducting surface of ITO-glass showed a slight increase in the intensity of its lowest energy Q-band after corona discharge as compared to the same before charging (Supplementary Fig. 33), indicating that there were certain electronic interactions between the charged particle and the π-unit that stabilised in this liquid. These small changes in the absorption spectra could be attributed to charging that occurred mainly at the surface of the film. A more precise analysis and fine-tuning of the electronic effect of corona-discharged π-unit are under investigation.

## Discussion

A novel strategy involving the use of a shielded π-unit of liquid porphyrins with trapped charge as an unprecedented ‘liquid-electret’ has been reported. The liquid-electret was primarily fabricated for demonstrating its piezoelectric and electroacoustic functions. Shielding of the π-core with hydrophobic and insulating bulky-alkyl chains enabled these liquid porphyrins to store electric charges, which was not possible with the unshielded solid H_2_TPP analogue. The fluidic nature of these liquids provided sufficient deformability that can be granted for device stretchability. This molecular design of an unconventional liquid electret presents a new direction toward mechanoelectrical and electroacoustic applications for advanced wearable/stretchable electronics. The fluidic nature of the liquid porphyrins was also studied and found to be influenced, slightly but surely, by the position of the alkyl substituents. Subtle porphyrin ring-distortions as the consequence of bulky-alkyl chains (in compounds **1**–**3**), which eventually had a negligible impact on the electronic perturbation, were unambiguously characterised by precise analyses of absorption, MCD, resonance Raman spectroscopy, and computational modelling. It is well known that the non-planar distortion of the porphyrinoid ring is invaluable to the biological functions of proteins. Therefore, this current finding could also be exploited to study the effect of conformational change on the biological functions associated with the porphyrinoid systems. A detailed understanding of the electronic structure of the shielded π-core environment is important for further design and implementation of liquid-electret devices toward wearable-healthcare electronic applications and such efforts are currently under progress in our laboratory.

## Methods

### Synthesis

Compounds **1–4** were synthesised via the typical porphyrin condensation reaction between pyrrole and the corresponding aldehydes at reflux for 4–5 h in propionic acid (Supplementary Fig. 1). It should be noted that the 2,5-derivatives (compounds **1**–**3**) existed as mixtures of all possible atropisomers at 296 K, which was confirmed by ^1^H NMR experiments. For example, the ^1^H NMR spectrum of compound **2** after refluxing in toluene overnight under argon was identical to the spectrum obtained without refluxing.

### MCD experiment

The solutions of compounds **1**–**5** (in CH_2_Cl_2_) in 1 cm quartz cuvettes were placed in the instrument holder sitting between two magnetic coils. As quartz is optically active, the same direction and orientation of the same quartz cell was maintained for all the experiments. In the case of solvent-free liquid state measurements, a thin film of the liquid sample was sandwiched between two quartz plates that were placed in a 2  mm quartz cuvette. This cuvette containing quartz plates was set in the sample holder through a spacer. In this case as well, the direction and orientation of both the cuvette and quartz plates were left unchanged for all measurements. Data acquisition for the Q-band envelopes needed nine-times accumulation to achieve sufficient S/N ratios because of their low intensity.

### RR measurements

A continuous wave (cw) single-mode diode laser (Innovative Photonic Solutions, I0405SD0050B-TK) with a wavelength of 405 nm was used to excite RR scattering of compounds **1**–**3**. Laser power at the sample point was 3.0 mW. The sample solution was placed in a glass tube used as a spinning cell and Raman light scattered at 90° was collected and focused onto the entrance slit of a spectrograph (HORIBA Jobin Ybon, iHR550) equipped with a CCD camera (Roper Scientific, SPEC-10:400B/LN-SN-U). The accumulation times for obtaining each spectrum were 10 and 5 min for 600–1100 and 1100–1650 cm^−1^ regions, respectively. The Raman shifts were calibrated against the Raman bands of cyclohexane and carbon tetrachloride. The calibration error was within 1 cm^−1^ for prominent bands.

### DFT calculations

The geometry optimisation and normal mode analysis were performed at the B3LYP/6-31 G* level. All the calculations were performed with Gaussian16 program package. A normal mode analysis was performed with DFT at the ground state equilibrium structure. To simplify the result spectra and avoid a high cost of calculation, a free-base porphyrin consisting of methoxy-substitutions at the 2,5-position of the *meso*-phenyl groups (compound **6**, Fig. 1a) was considered as the model compound. Assignments of the normal modes of compound **6** predicted by the DFT calculations were made based on the symmetry and the calculated frequencies agreed well with the experimentally observed frequencies (Supplementary Table 2).

### Electret device fabrication

A liquid porphyrin film (with compound **2**) was formed on a glass substrate with patterned ITO electrodes by blade coating (Supplementary Fig. 29). Because of the high viscosity of the liquid porphyrin, no repellence was observed between the ITO substrate and liquid sample and a uniform film was formed. This film was charged through a corona discharge process. In the corona discharge process, an electrical discharge gun (Green Techno Co Ltd., GC-80N) which was connected to a voltage source was used for the ionisation of gas molecules in air. The film was placed on a hot plate (at 393 K) which was set below the corona-discharge gun at a distance of 30 cm. The corona discharge process was run for 30 min with applied voltage of −6.8 kV at 393 K. The charged gas ions (HO^−^, NO_2_^−^, NO_3_^−^, etc.) generated by corona discharge were flown down to the surface of the liquid porphyrin film, where they were trapped. Subsequently, the entire substrate was heated for further promoting charge injection into the porphyrin core. During the corona discharge process, liquid porphyrin gradually became charged and moved to the bottom electrodes that were connected to the ground, indicating their charged-state characteristics.

## Data availability

The data that support the finding of this study are available from the corresponding author on reasonable request.

## Supplementary information

Supplementary Information

Description of Additional Supplementary Files

Supplementary Movie 1

## References

[1] Xu S, Hansen BJ, Wang ZL (2010). Piezoelectric-nanowire-enabled power source for driving wireless microelectronics. Nat. Commun..

[2] Chung SY (2012). All-solution-processes flexible thin film piezoelectric nanogenerator. Adv. Mater..

[3] Sessler, G. M. & Gerhard-Multhaupt, R. *Electrets*. Vols. I–II (Laplacian, Morgan Hill, 1999).

[4] Jacobs HO, Whitesides GM (2001). Submicrometer patterning of charge in thin-film electrets. Science.

[5] Sessler GM (2001). Electrets: recent developments. J. Electrostat..

[6] Gerhard-Multhaupt R (2002). Less can be more. Holes in polymers lead to a new paradigm of piezoelectric materials for electret transducers. IEEE Trans. Dielectr. Electr. Insulation.

[7] Chou Y-H, Chang H-C, Liu C-L, Chen W-C (2015). Polymeric charge storage electrets for non-volatile organic field effect transistor memory devices. Polym. Chem..

[8] Grimm RL, Beauchamp JL (2003). Field-induced droplet ionization mass spectrometry. J. Phys. Chem. B.

[9] Mettu S, Berry JD, Dagastine RR (2017). Charge and film drainage of colliding oil drops coated with the nonionic surfactant C_12_E_5_. Langmuir.

[10] Beattie JK, Djerdjev AM (2004). The pristine oil/water interface: surfactant-free hydroxide-charged emulsions. Angew. Chem. Int. Ed..

[11] Zhu J, Song W, Ma F, Wang H (2018). A flexible multi-layer electret nanogenerator for bending deformation energy harvesting and strain sensing. Mater. Res. Bull..

[12] Chen S (2018). Noncontact heartbeat and respiration monitoring based on a hollow microstructured self-powered pressure sensor. ACS Appl. Mater. Interfaces.

[13] Schroeder TBH (2017). An electric-eel-inspired soft power source from stacked hydrogels. Nature.

[14] Ghosh A, Nakanishi T (2017). Frontiers of solvent-free functional molecular liquids. Chem. Commun..

[15] Marco AB (2017). Twisted aromatic frameworks: readily exfoliable and solution processable two-dimensional conjugated microporous polymers. Angew. Chem. Int. Ed..

[16] Fukui N, Kim T, Kim D, Osuka A (2017). Porphyrin arch-tapes: synthesis, contorted structures, and full conjugation. J. Am. Chem. Soc..

[17] Rickhaus M (2017). Single-acetylene linked porphyrin nanorings. J. Am. Chem. Soc..

[18] Richert S (2017). Constructive quantum interference in a bis-copper six-porphyrin nanoring. Nat. Commun..

[19] Shi Z, Franco R, Haddad R, Shelnutt JA, Ferreira GC (2006). The conserved active-site loop residues of ferrochelatase induce porphyrin conformational changes necessary for catalysis. Biochemistry.

[20] Chen S (2018). Noncontact heartbeat and respiration monitoring based on a hollow microstructured self-powered pressure sensor. ACS Appl. Mater. Interfaces.

[21] Fan FR, Tang W, Wang ZL (2016). Flexible nanogenerators for energy harvesting and self-powered electronics. Adv. Mater..

[22] Guerbet M (1909). Condensation of isopropyl alcohol with its sodium compound: formation of methylisobutylcarbinol and 2,4-dimethylheptanol-6. C. R. Chim..

[23] Lu F (2017). A guide to design functional molecular liquids with tailorable properties using pyrene-fluorescence as a probe. Sci. Rep..

[24] Li H (2013). Alkylated-C_60_ based soft materials: regulation of self-assembly and optoelectronic properties by chain branching. J. Mater. Chem. C.

[25] Saeki A, Seki S, Takenobu T, Iwasa Y, Tagawa S (2008). Mobility and dynamics of charge carriers in rubrene single crystals studied by flash-photolysis microwave conductivity and optical spectroscopy. Adv. Mater..

[26] Hizume Y (2010). Chiroselective Assembly of a chiral porphyrin-fullerene dyad: photoconductive nanofiber with a top-class ambipolar charge-carrier mobility. J. Am. Chem. Soc..

[27] Narayan B (2018). The effect of regioisomerism on the photophysical properties of alkylated-naphthalene liquids. Phys. Chem. Chem. Phys..

[28] Saito H, Tuzi S, Yamaguchi S, Tanio M, Naito A (2000). Conformation and backbone dynamics of bacteriorhodopsin revealed by ^13^C-NMR. Biochim. Biophys. Acta.

[29] Yamamuro O, Oguni M, Matsuo T, Suga H (1987). Construction of an adiabatic high-pressure calorimeter using helium gas for pressurization. Bull. Chem. Soc. Jpn.

[30] Chang SS, Bestul AB (1972). Heat capacity and thermodynamic properties of *o*-terphenyl crystal, glass and liquid. J. Chem. Phys..

[31] Maruyama S, Sato K, Iwahashi H (2010). Room temperature liquid porphyrins. Chem. Lett..

[32] Nowak-Krόl A, Gryko D, Gryko DT (2010). *Meso*-substitute liquid porphyrins. Chem. Asian J..

[33] Chino Y (2017). Stimuli-responsive rheological properties for liquid phthalocyanines. Chem. Lett..

[34] Gouterman M (1961). Spectra of porphyrins. J. Mol. Spectrosc..

[35] Gouterman M, Wagnière GH (1963). Spectra of porphyrins, part II. Four orbital model. J. Mol. Spectrosc..

[36] Michl J (1978). Magnetic circular dichroism of cyclic *π*-electron systems. 1. Algebraic solution of the perimeter model for the *A* and *B* terms of high-symmetry systems with a (4*N* + 2)-electron [*n*]annulene perimeter. J. Am. Chem. Soc..

[37] Michl J (1980). Electronic structure of aromatic *π*-electron systems as reflected in their MCD spectra. Pure Appl. Chem..

[38] Mack J, Asano Y, Kobayashi N, Stillman MJ (2005). Application of MCD spectroscopy and TD−DFT to a highly non-planar porphyrinoid ring system. New insights on red-shifted porphyrinoid spectral bands. J. Am. Chem. Soc..

[39] Keegan JD (1982). Magnetic circular dichroism studies. 60. Substituent-induced sign variation in the magnetic circular dichroism spectra of reduced porphyrins. 1. Spectra and band assignments. J. Am. Chem. Soc..

[40] Keegan JD (1982). Magnetic circular dichroism studies. 61. Substituent-induced sign variation in the magnetic circular dichroism spectra of reduced porphyrins. 2. Perturbed molecular orbital analysis. J. Am. Chem. Soc..

[41] Djerassi C (1984). Sign variation in the magnetic circular dichroism spectra of free-base porphyrins having a single *π*-acceptor pyrrole ring substituent. Structure implications^1^. J. Am. Chem. Soc..

[42] Michl J (1978). Magnetic circular dichroism of cyclic *π*-electron systems. 2. Algebraic solution of the perimeter model for the *B* terms of systems with a (4*N* + 2)-electron [*n*]annulene perimeter. J. Am. Chem. Soc..

[43] Venkateshrao S, Yin J, Jarzeücki AA, Schultz PG, Spiro TG (2004). Porphyrin distortion during affinity maturation of a ferrochelatase antibody, monitored by resonance Raman spectroscopy. J. Am. Chem. Soc..

[44] Saini GS (2006). Resonance Raman study of free-base tetraphenylporphine and its dication. Spectrochim. Acta A.

[45] Spiro, T. G, Li, X.-Y. *Biological Application of Raman Spectroscopy*. Spiro, T. G. Ed., Vol. III, p. 1–37 (John Wiley and Sons, New York, 1988).

[46] Ryeng H, Ghosh A (2002). Do nonplanar distortions of porphyrins bring about strongly red-shifted electronic spectra? Controversy, consensus, new developments, and relevance to chelatases. J. Am. Chem. Soc..

[47] Ravikanth M, Chandrashekar TK (1995). Nonplanar porphyrins and their biological relevance: ground and excited state dynamics. Struct. Bonding.

[48] Shelnutt JA (1998). Nonplanar porphyrins and their significance in proteins. Chem. Soc. Rev..

[49] Wu N (2018). Theoretical study and structural optimization of a flexible piezoelectret-based pressure sensor. J. Mater. Chem. A.

[50] Nobeshima T, Uemura S, Yoshida M, Kamata T (2016). Stretchable conductor from oriented short conductive fibers for wiring soft electronics. Polym. Bull..

