# Supplementary material for: Soft chromophore featured liquid porphyrins and their utilization toward liquid electret applications
Source: Nat Commun. 2019 Sep 30;10:4210. doi: 10.1038/s41467-019-12249-8 (PMC6768991; doi:10.1038/s41467-019-12249-8)
Supplement: Supplementary file 1 — Supplementary Information [file 41467_2019_12249_MOESM1_ESM.pdf]

Supplementary Information

Soft chromophore featured liquid porphyrins and their utilization toward liquid electret applications

Ghosh *et al*.

Contents:

Supplementary Figures

Supplementary Figure 1….. ….. ….. ….. ….. ….. ….. ….. 4

Supplementary Figure 2….. ….. ….. ….. ….. ….. ….. ….. 5

Supplementary Figure 3….. ….. ….. ….. ….. ….. ….. ….. 6

Supplementary Figure 4….. ….. ….. ….. ….. ….. ….. ….. 7

Supplementary Figure 5….. ….. ….. ….. ….. ….. ….. ….. 8

Supplementary Figure 6….. ….. ….. ….. ….. ….. ….. ….. 9

Supplementary Figure 7….. ….. ….. ….. ….. ….. ….. ….. 10

Supplementary Figure 8….. ….. ….. ….. ….. ….. ….. ….. 11

Supplementary Figure 9….. ….. ….. ….. ….. ….. ….. ….. 12

Supplementary Figure 10….. ….. ….. ….. ….. ….. ….. ….. 13

Supplementary Figure 11….. ….. ….. ….. ….. ….. ….. ….. 14

Supplementary Figure 12….. ….. ….. ….. ….. ….. ….. ….. 15

Supplementary Figure 13….. ….. ….. ….. ….. ….. ….. ….. 16

Supplementary Figure 14….. ….. ….. ….. ….. ….. ….. ….. 17

Supplementary Figure 15….. ….. ….. ….. ….. ….. ….. ….. 18

Supplementary Figure 16….. ….. ….. ….. ….. ….. ….. ….. 19

Supplementary Figure 17….. ….. ….. ….. ….. ….. ….. ….. 20

Supplementary Figure 18….. ….. ….. ….. ….. ….. ….. ….. 21

Supplementary Figure 19….. ….. ….. ….. ….. ….. ….. ….. 22

Supplementary Figure 20….. ….. ….. ….. ….. ….. ….. ….. 23

Supplementary Figure 21….. ….. ….. ….. ….. ….. ….. ….. 24

Supplementary Figure 22….. ….. ….. ….. ….. ….. ….. ….. 25

Supplementary Figure 23….. ….. ….. ….. ….. ….. ….. ….. 26

Supplementary Figure 24….. ….. ….. ….. ….. ….. ….. ….. 27

Supplementary Figure 25….. ….. ….. ….. ….. ….. ….. ….. 28

Supplementary Figure 26….. ….. ….. ….. ….. ….. ….. ….. 29
Supplementary Figure 27….. ….. ….. ….. ….. ….. ….. ….. 30
Supplementary Figure 28….. ….. ….. ….. ….. ….. ….. ….. 31
Supplementary Figure 29….. ….. ….. ….. ….. ….. ….. ….. 32
Supplementary Figure 30….. ….. ….. ….. ….. ….. ….. ….. 33
Supplementary Figure 31….. ….. ….. ….. ….. ….. ….. ….. 34
Supplementary Figure 32….. ….. ….. ….. ….. ….. ….. ….. 35
Supplementary Figure 33….. ….. ….. ….. ….. ….. ….. ….. 36

Supplementary Tables

Supplementary Table 1….. ….. ….. ….. ….. ….. ….. ….. 37
Supplementary Table 2….. ….. ….. ….. ….. ….. ….. ….. 38
Supplementary Table 3….. ….. ….. ….. ….. ….. ….. ….. 39
Supplementary Table 4….. ….. ….. ….. ….. ….. ….. ….. 40

Supplementary Notes

Supplementary Note 1: MCD spectral analysis….. ….. ….. ….. ….. 41–42
Supplementary Note 2: Assignments of RR bands….. ….. ….. ….. 43–44

Supplementary Methods

Chemical….. ….. ….. ….. ….. ….. ….. ….. ….. ….. 44
Instrumentation….. ….. ….. ….. ….. ….. ….. ….. ….. 44–46
Synthesis….. ….. ….. ….. ….. ….. ….. ….. ….. ….. 46–48

Supplementary References….. ….. ….. ….. ….. ….. ….. 49–50

Supplementary Figures

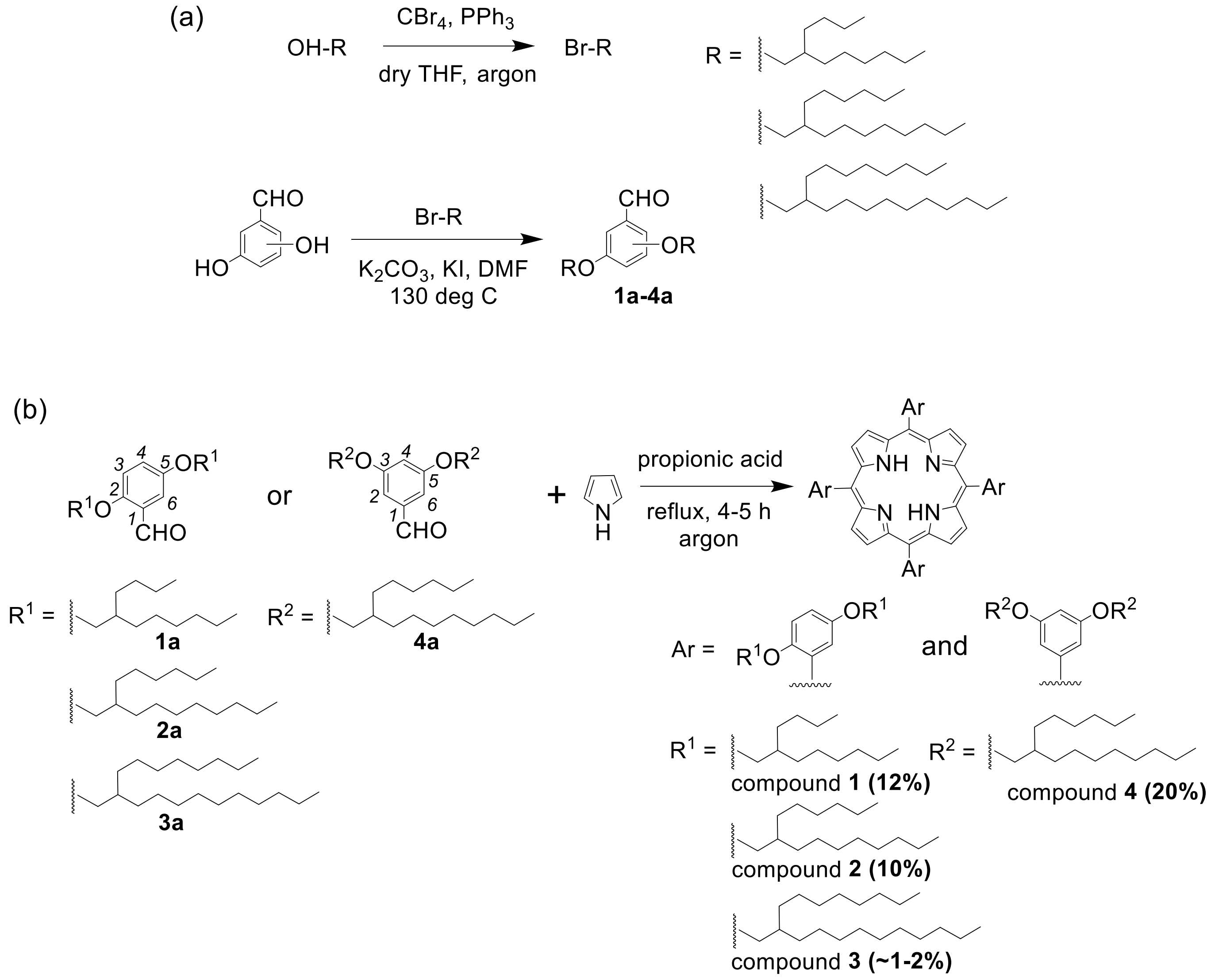

Supplementary Figure 1. Synthetic scheme. (a) Compounds **1–4** and (b) corresponding precursors (**1a–4a**). See the section 'Synthesis' (Supplementary Methods) for detail synthetic methods and characterizations. The yields of compounds **1–4** are provided in the parenthesis. The numbering of 2,5- and 3,5- positions are considered with respect to the phenyl carbon which is attached to the *meso*-carbon of the porphyrin ring.

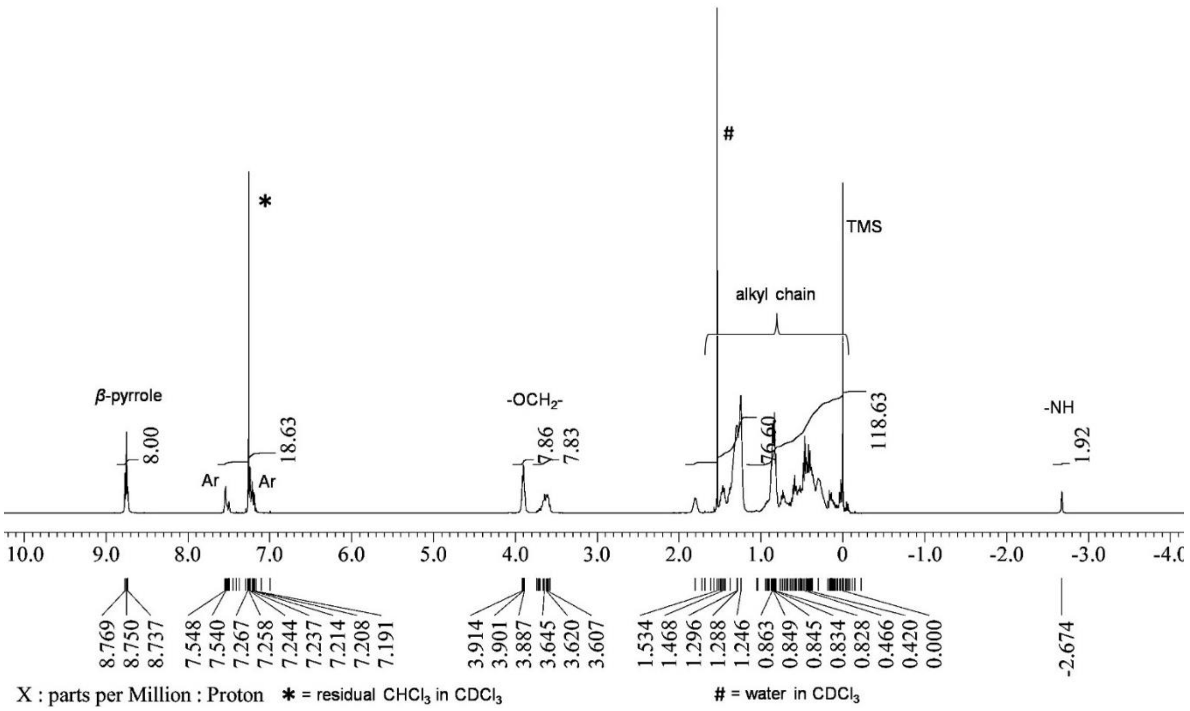

Supplementary Figure 2. ^{1}H NMR Spectrum. ^{1}H NMR spectrum of compound **1** recorded in $CDCl_3$ at 296 K.

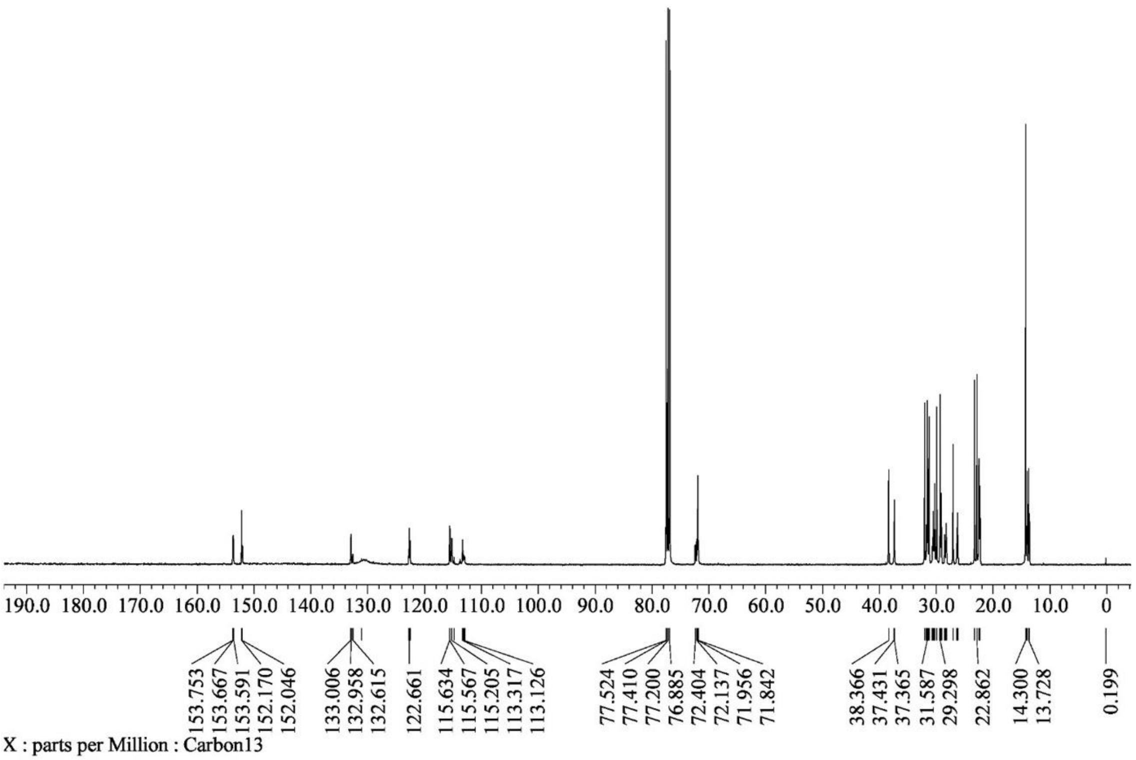

Supplementary Figure 3. ^{13}C NMR Spectrum. ^{13}C NMR spectrum of compound **1** recorded in $CDCl_3$ at 296 K.

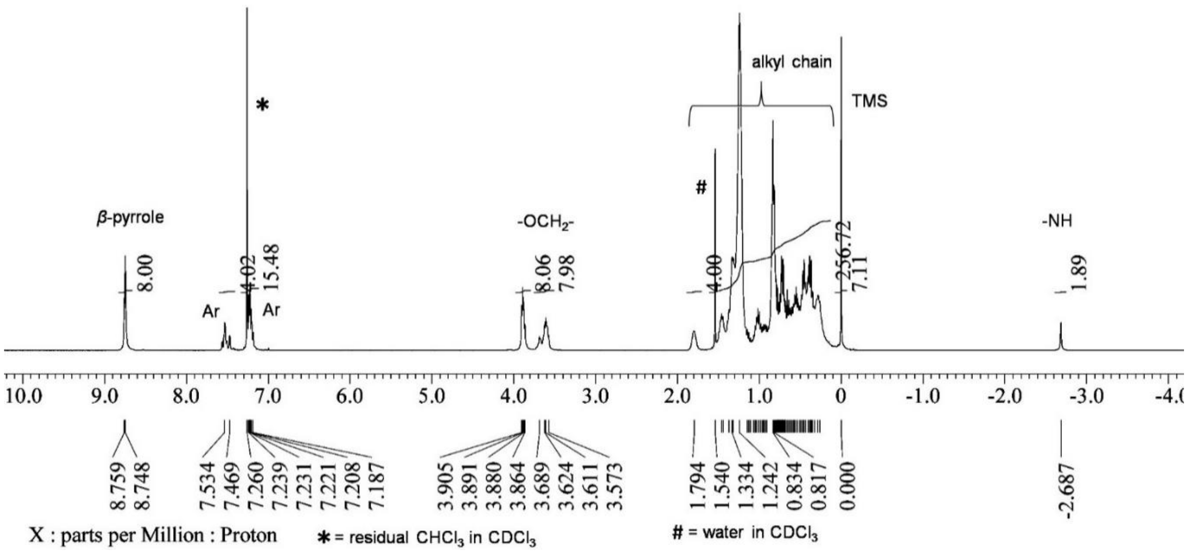

Supplementary Figure 4. ^{1}H NMR Spectrum. ^{1}H NMR spectrum of compound **2** recorded in $CDCl_3$ at 296 K.

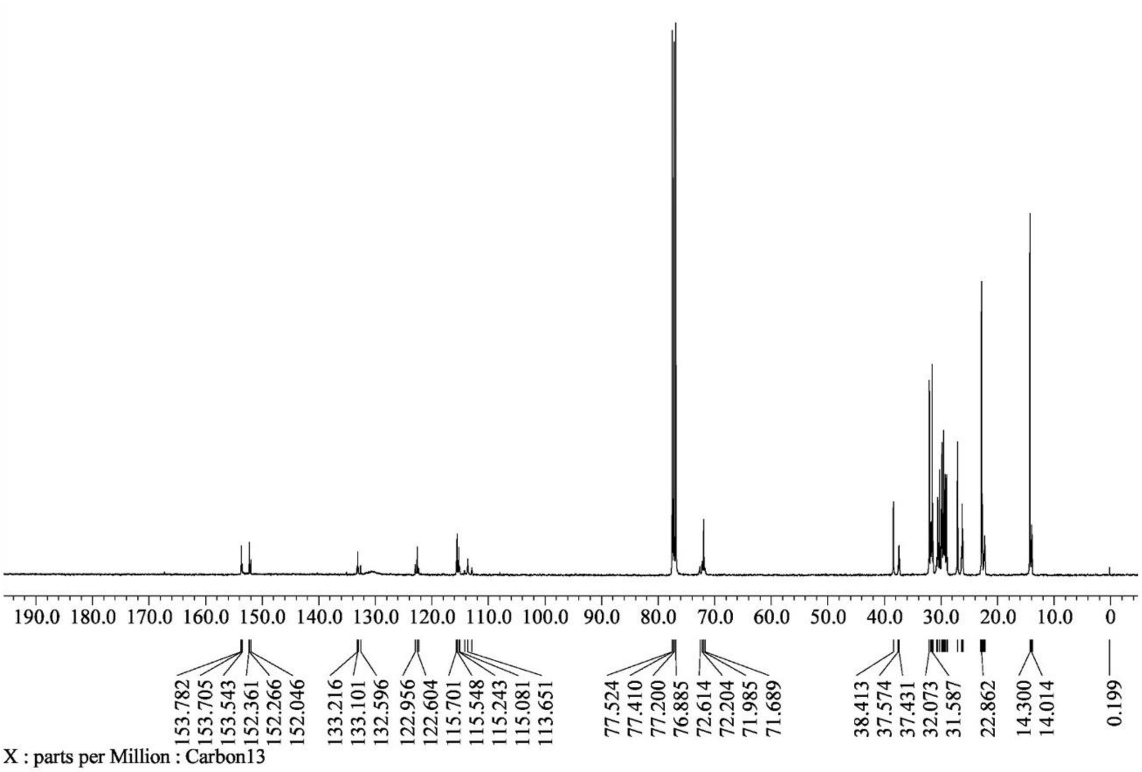

Supplementary Figure 5. ^{13}C NMR Spectrum. ^{13}C NMR spectrum of compound **2** recorded in $CDCl_3$ at 296 K.

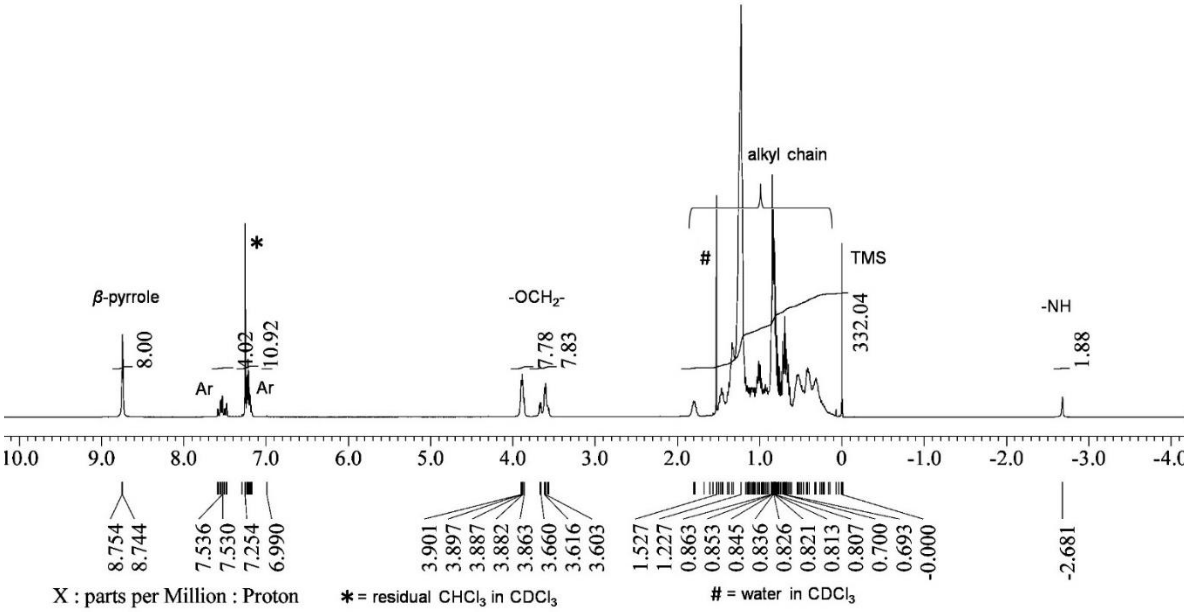

Supplementary Figure 6. ^{1}H NMR Spectrum. ^{1}H NMR spectrum of compound **3** recorded in $CDCl_3$ at 296 K.

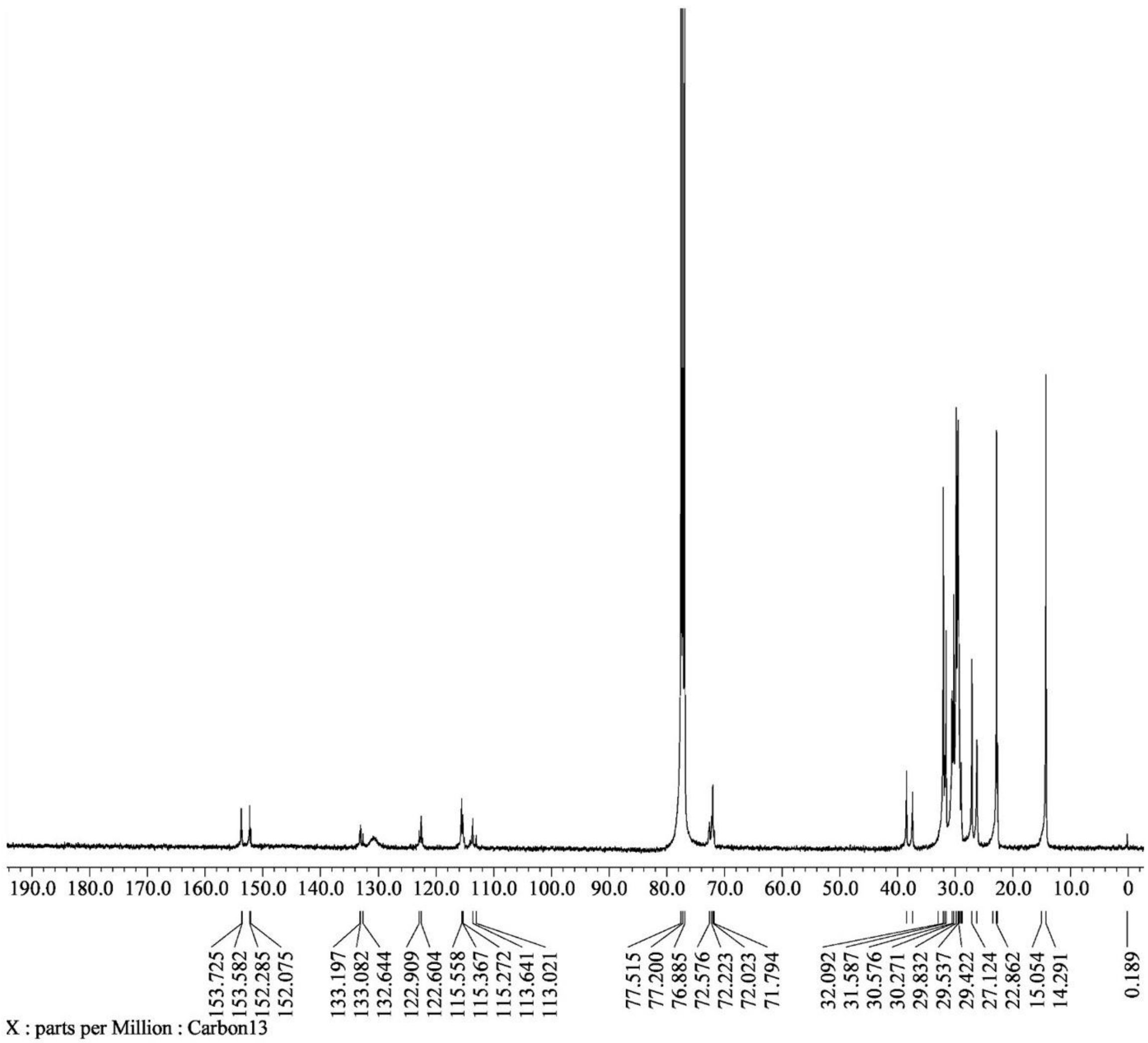

Supplementary Figure 7. ^{13}C NMR Spectrum. ^{13}C NMR spectrum of compound **3** recorded in $CDCl_3$ at 296 K.

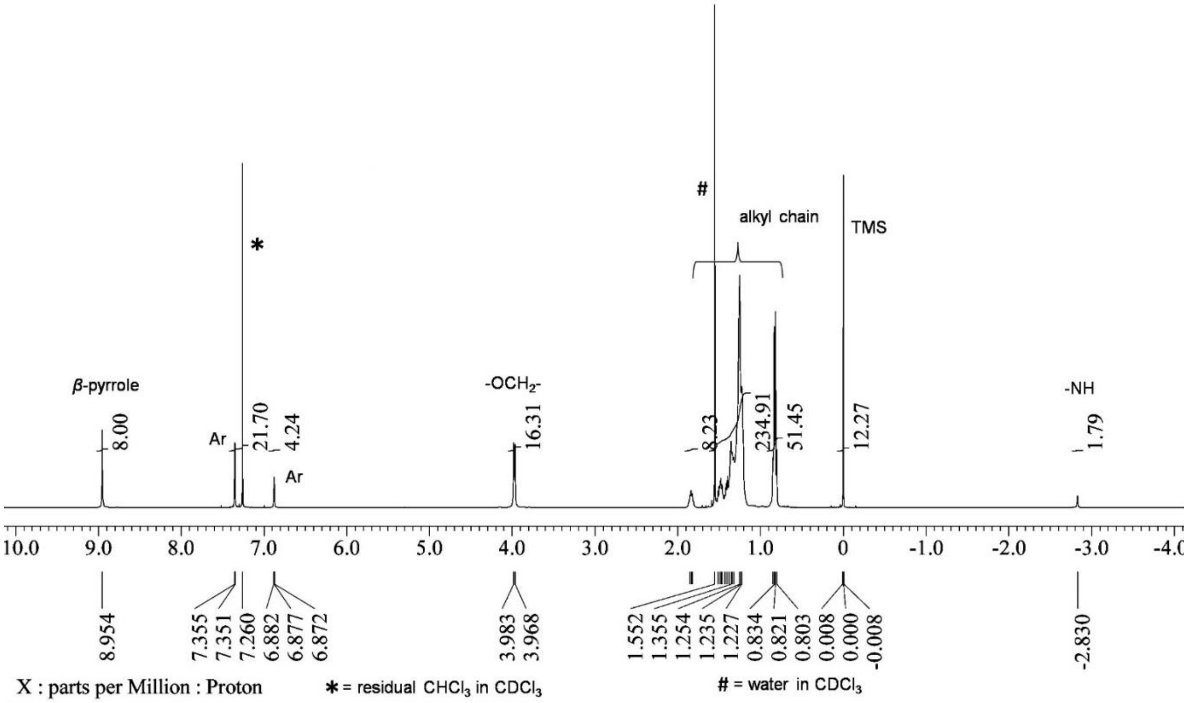

Supplementary Figure 8. ^{1}H NMR Spectrum. ^{1}H NMR spectrum of compound **4** recorded in $CDCl_3$ at 296 K.

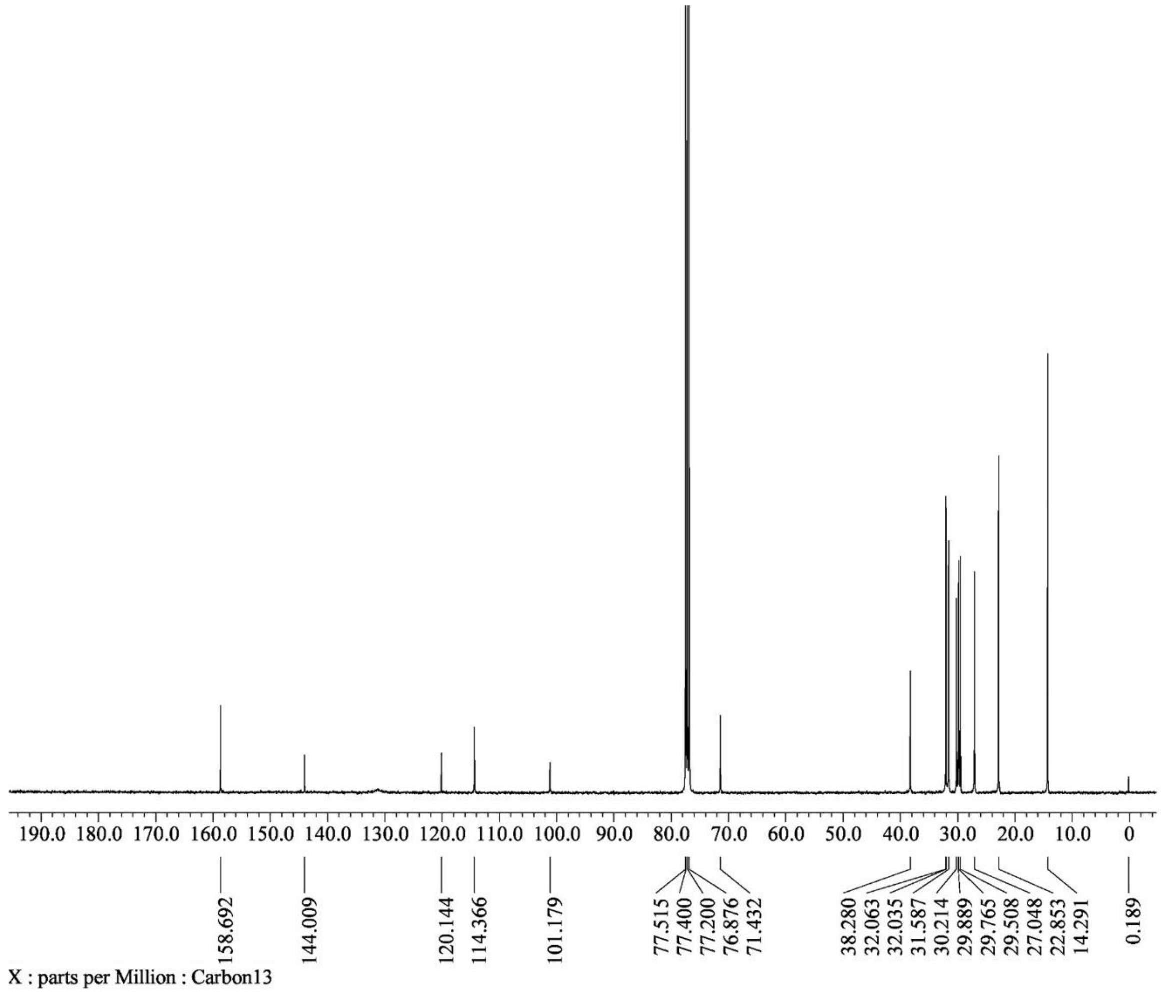

Supplementary Figure 9. ^{13}C NMR Spectrum. ^{13}C NMR spectrum of compound **4** recorded in $CDCl_3$ at 296 K.

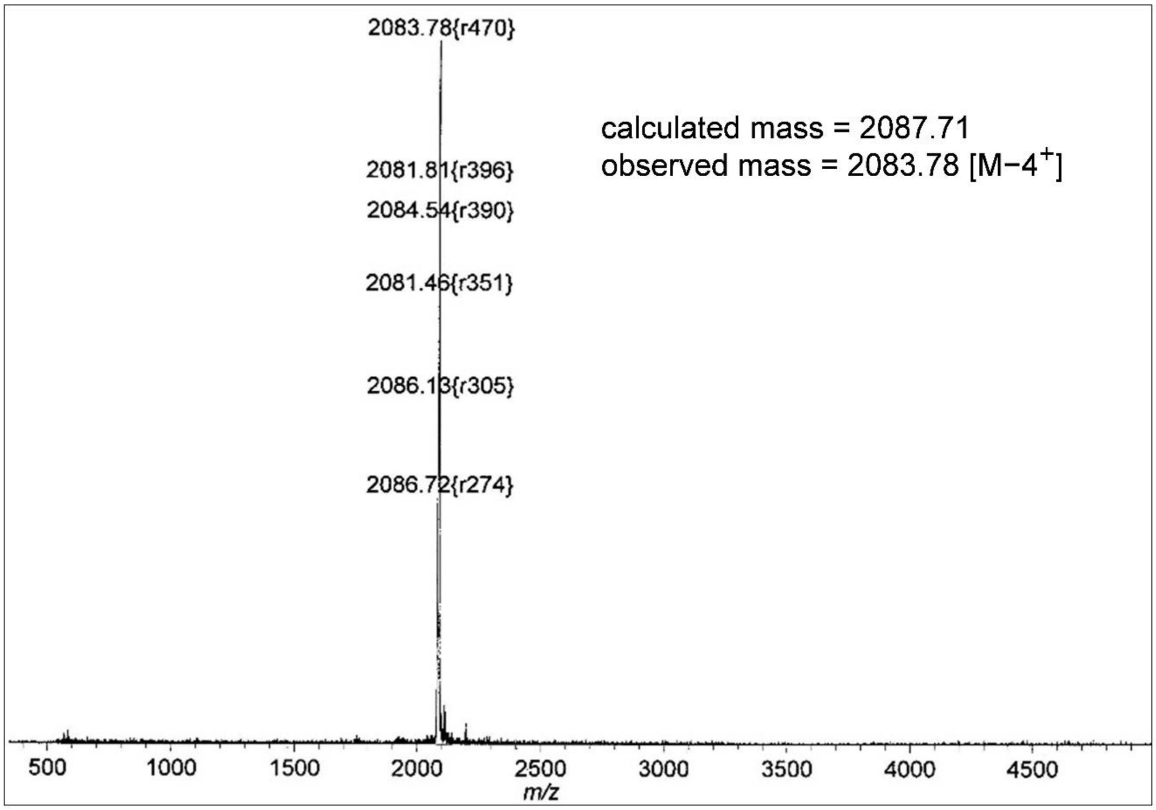

Supplementary Figure 10. MALDI-TOF mass spectrum. MALDI-TOF mass spectrum of compound **1** recorded by using 4′-Hydroxyazobenzene-2-carboxylic acid (HABA) as matrix.

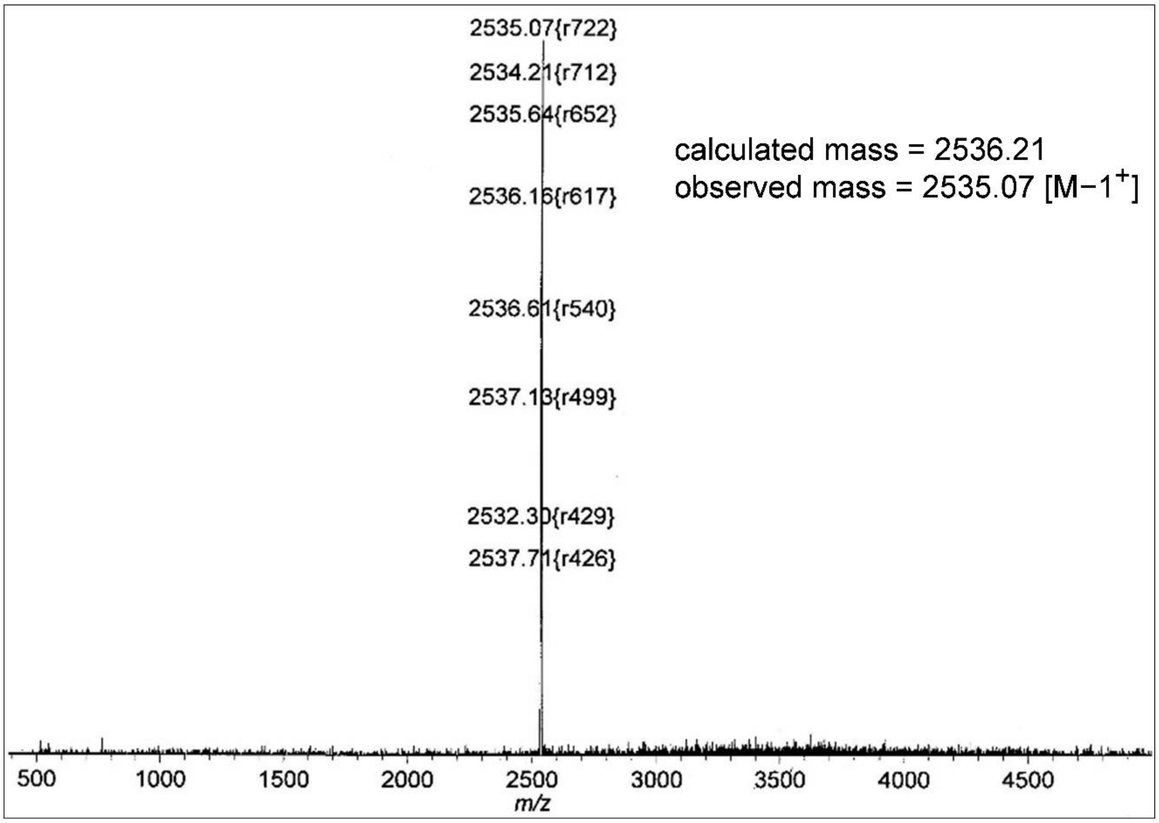

Supplementary Figure 11. MALDI-TOF mass spectrum. MALDI-TOF mass spectrum of compound **2** recorded by using 4′-Hydroxyazobenzene-2-carboxylic acid (HABA) as matrix.

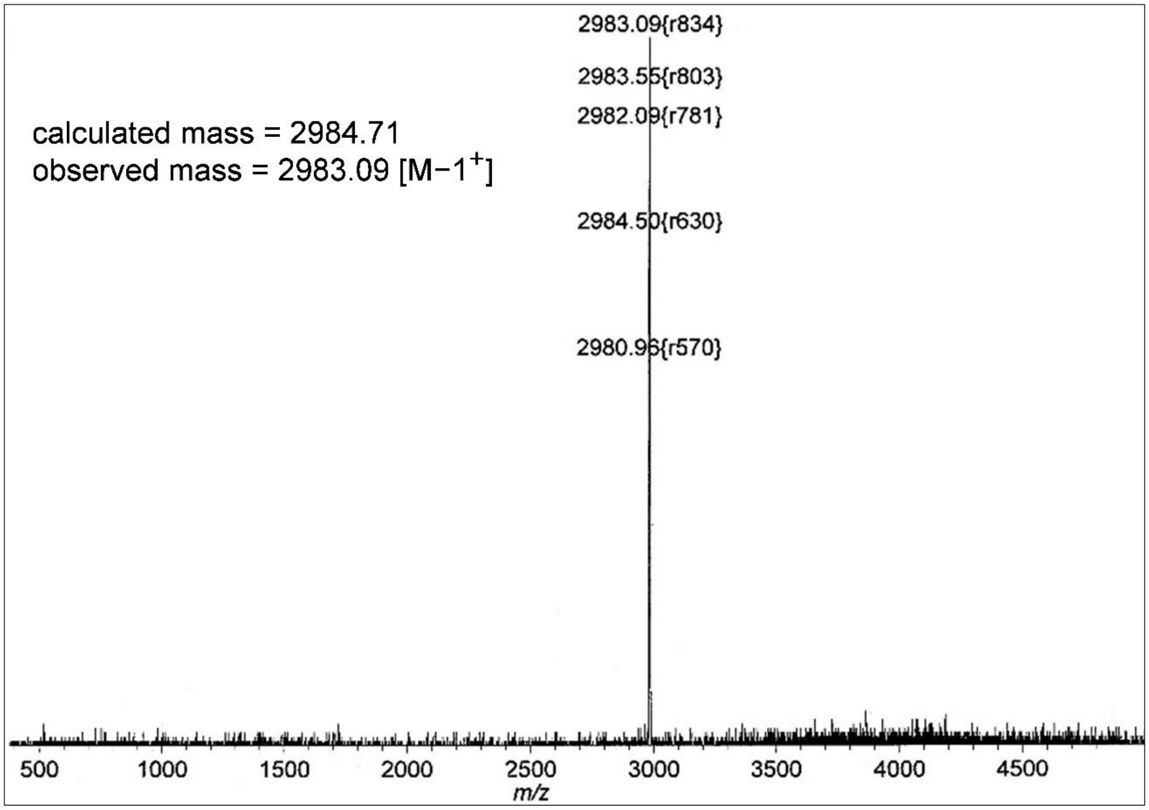

Supplementary Figure 12. MALDI-TOF mass spectrum. MALDI-TOF mass spectrum of compound **3** recorded by using 4′-Hydroxyazobenzene-2-carboxylic acid (HABA) as matrix.

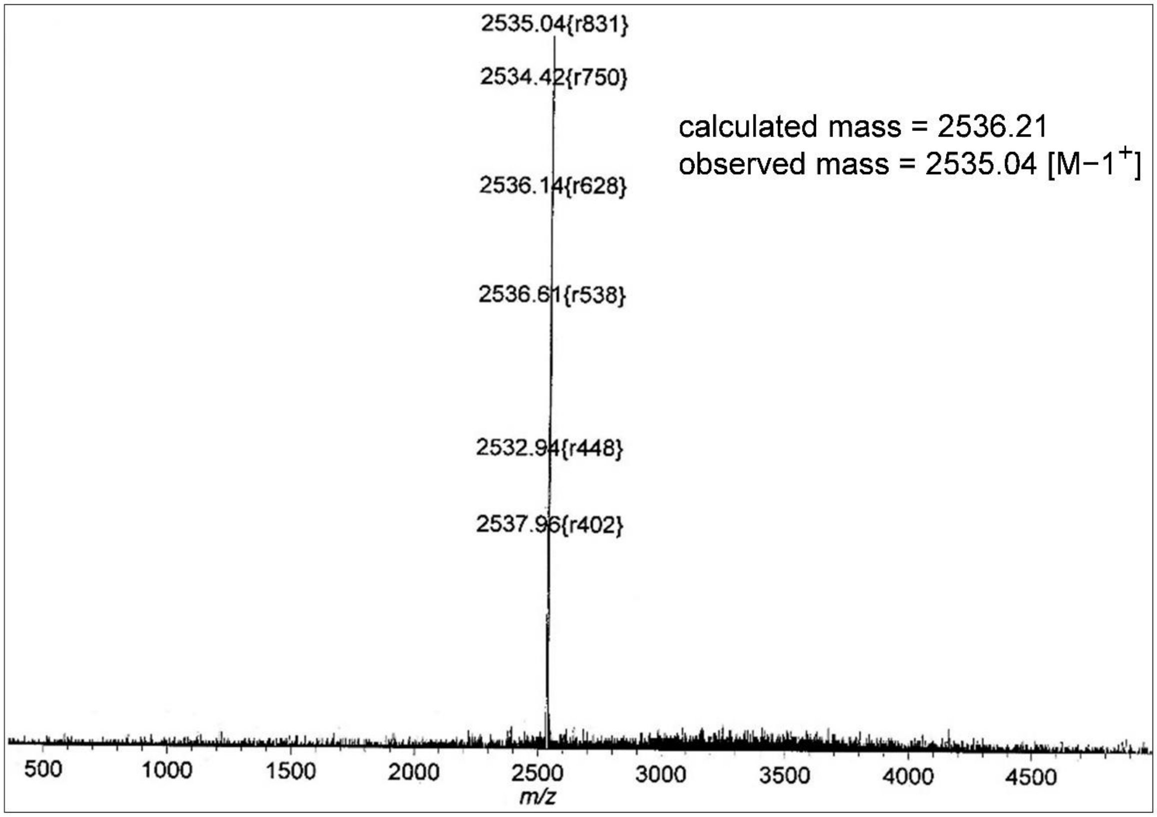

Supplementary Figure 13. MALDI-TOF mass spectrum. MALDI-TOF mass spectrum of compound **4** recorded by using 4′-Hydroxyazobenzene-2-carboxylic acid (HABA) as matrix.

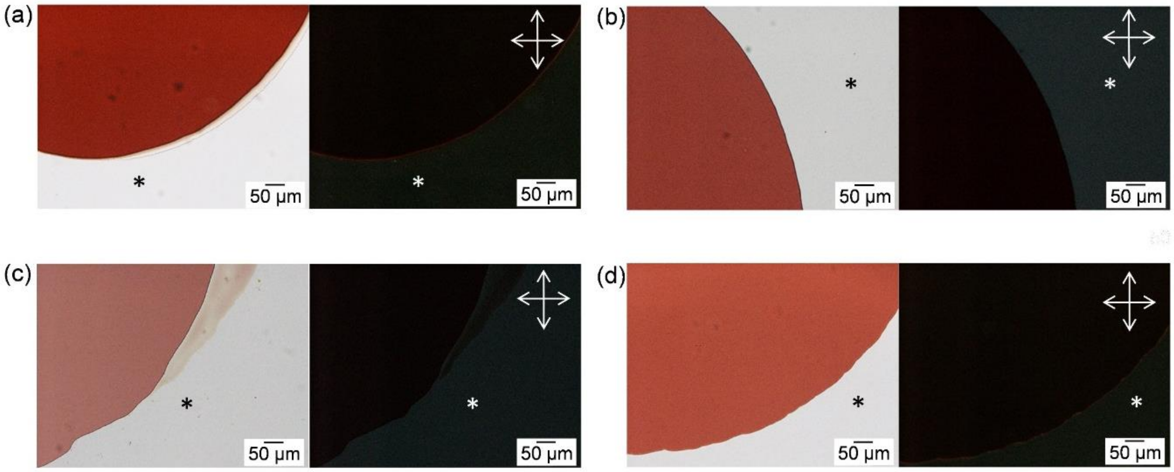

Supplementary Figure 14. Optical microscopy properties. Optical microscopy images with/without polarizer of compounds **1–4** (a–d, respectively) in their solvent-free liquid state at 296 K. The corresponding dark microscope images were observed under cross polarized light. The '*' represents the space without samples. The reddish-brown colour represent the liquids which was found to flow slowly on the glass surface.

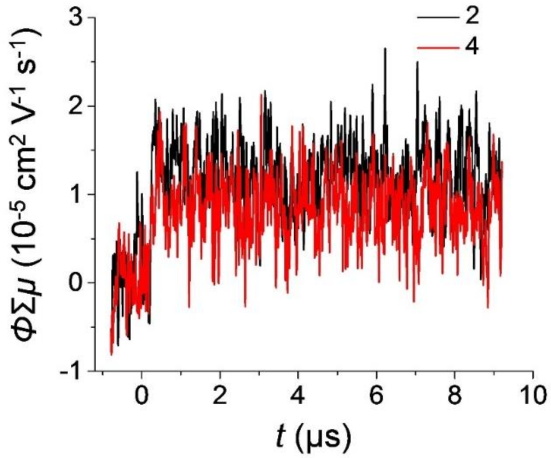

Supplementary Figure 15. Photoconductivity analysis. Comparison of FP-TRMC profiles of compounds **2** and **4** recorded at 296 K, upon excitation with a 355 nm laser pulse. The absence of any distinct value of photoconductivity ($\phi\Sigma\mu_{max}$) indicating the disorder-amorphous nature of both liquids. $\phi\Sigma\mu$ = transient photoconductivity, ϕ represents the quantum efficiency of the charge carrier generation and $\Sigma\mu$ is the sum of the nanometre-scale charge carrier mobilities. The photoconductivity maximum ($\phi\Sigma\mu_{max}$) values observed for compounds **2** and **4** were 1.6×10^{-5} cm^2 V^{-1} s^{-1} and 1.1×10^{-5} cm^2 V^{-1} s^{-1}, respectively.

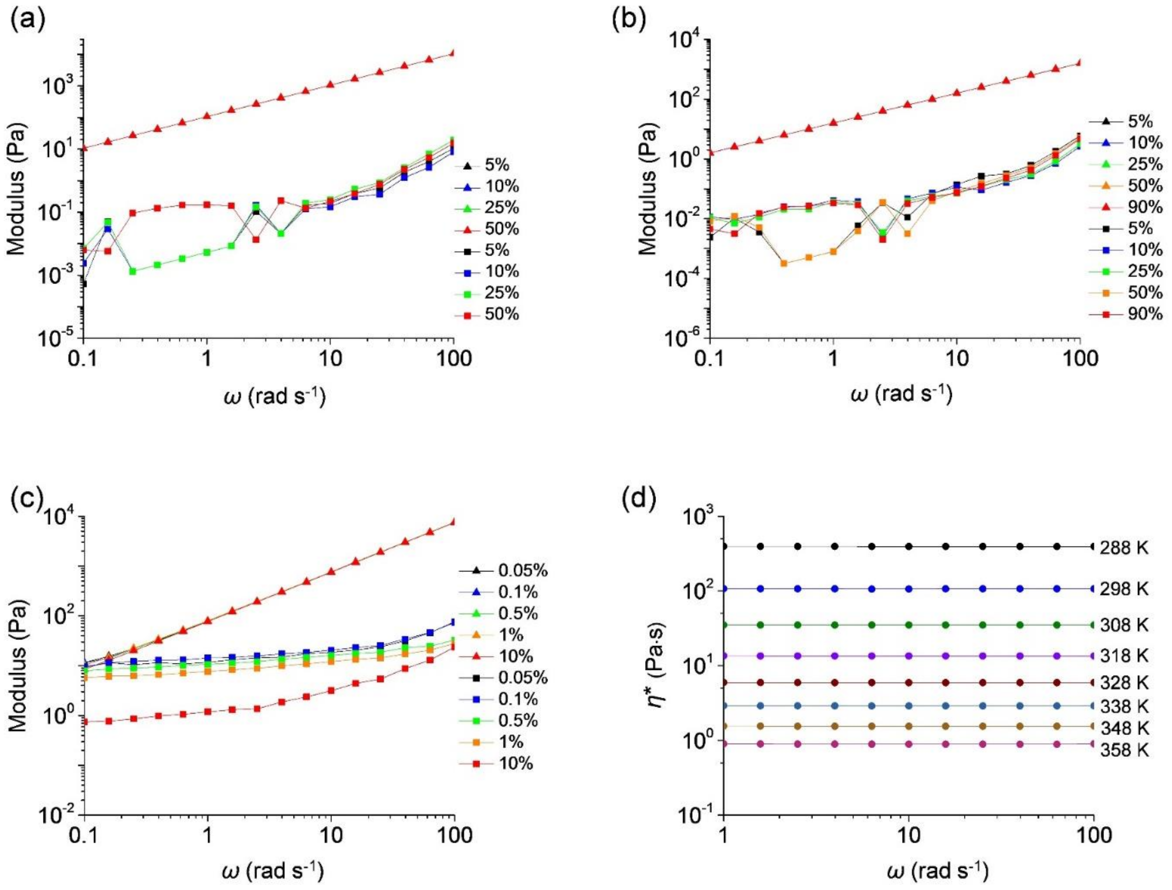

Supplementary Figure 16. Viscoelastic fluidic properties. Change of modulus (G' and G'') as a function of angular frequency (ω) at various strain amplitude (γ%) collected for (a) compound **1**, (b) compound **2** and (c) compound **4** at 296 K. G' and G'' are represented with the symbols '■' and '▲' respectively. In all cases $G'' > G'$, indicating their liquid character at 296 K. (d) Change in the complex viscosity (η*) of compound **4** as a function of ω at various temperature. Although both compounds **1** and **2** behaved as Newtonian fluid, the compound **1** exhibited one order higher magnitude of η*, 106.9 Pa·s at 298 K and ω = 25.1 rad s^{-1} than compound **2**. The higher viscosity of compound **1** was probably because of shorter alkyl chain which reduced the alkyl content in the molecule and strengthen the π–π interactions.

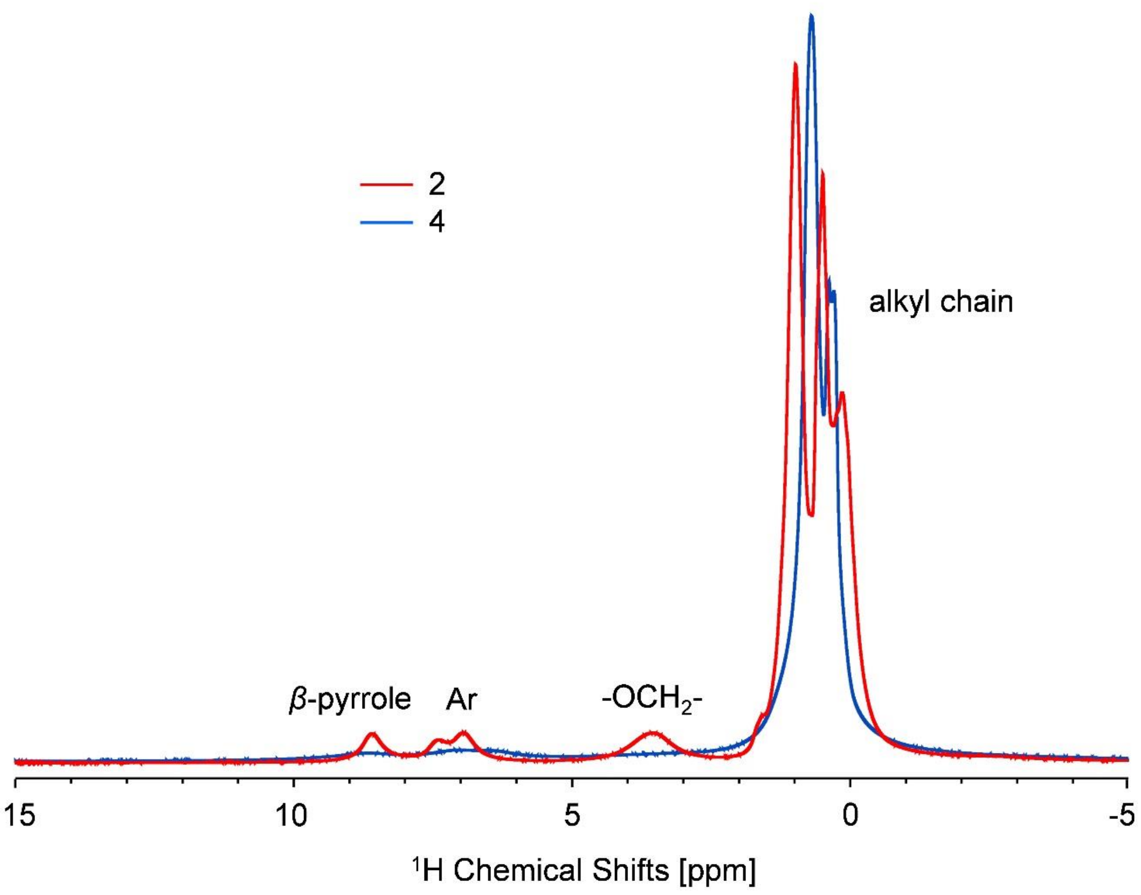

Supplementary Figure 17. ^{1}H NMR spectra of solvent free liquids. Comparison of magic-angle spinning (MAS) ^{1}H NMR spectra for solvent-free state of compounds **2** and **4** at 13.5 kHz, 296 K. Relatively sharp and featured signals were observed for compound **2**.

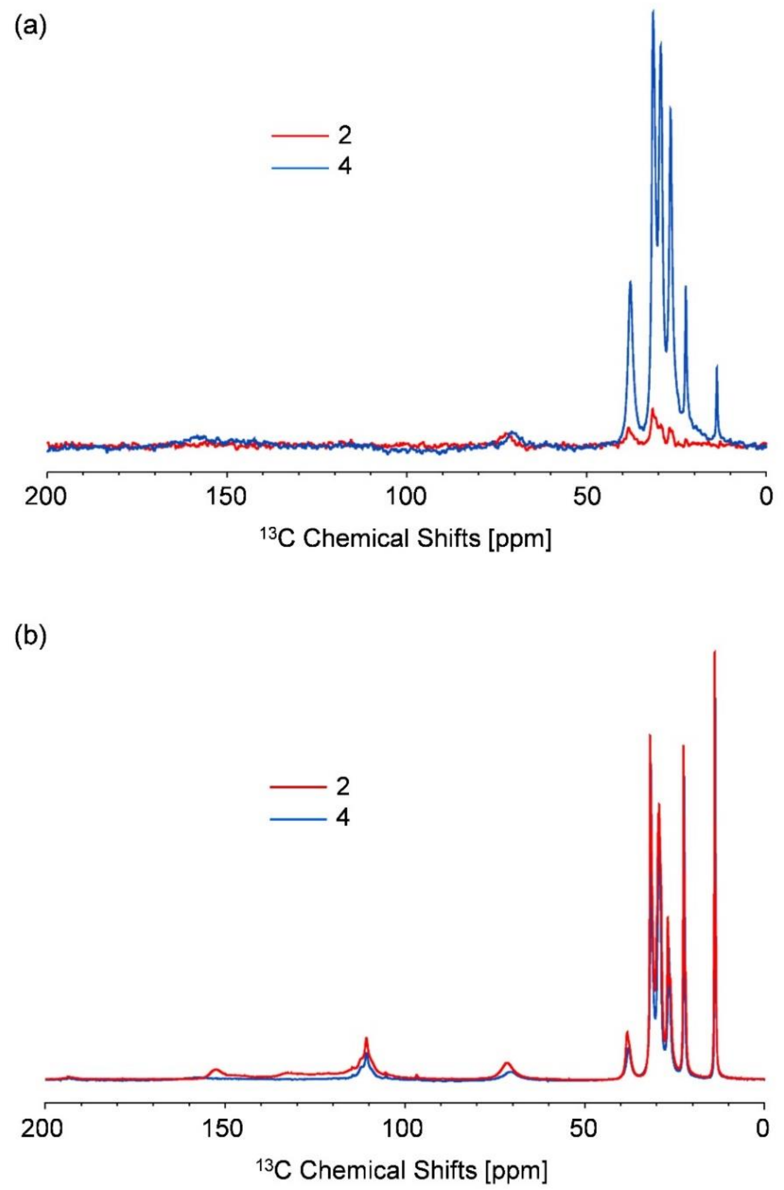

Supplementary Figure 18. ^{13}C NMR spectra of solvent free liquids. Comparison of full spectra of magic-angle spinning (MAS) ^{13}C NMR for solvent-free state between compounds **2** and **4** under 12.5 kHz at 296 K. In the CP-MAS mode (a), compound **4** exhibited much sharper, intense and featured signals while in DD-MAS mode (b) compound **2** showed relatively sharp and intense signal. Sharp and intense signals in DD-MAS indicate flexible motion of molecules with shorter correlation time, while the rigid molecules with longer correlation time exhibit sharper and intense signal in CP-MAS. Therefore, these solid-state NMR results revealed higher molecular motion with shorter correlation time of compound **2** over compound **4**.

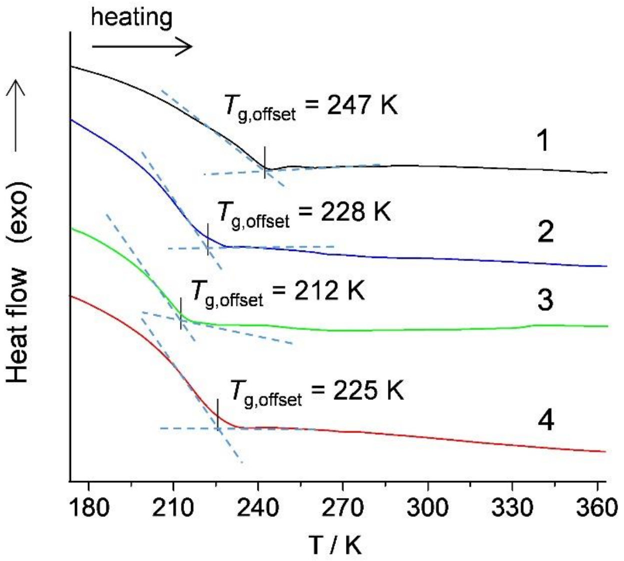

Supplementary Figure 19. Thermal properties of liquids. DSC thermograms in the heating trace of compounds **1–4** recorded under nitrogen atmosphere at the scan rate of 10 °C min^{-1}. The offset glass-transition temperature ($T_{g,offset}$) are shown in the corresponding DSC curves.

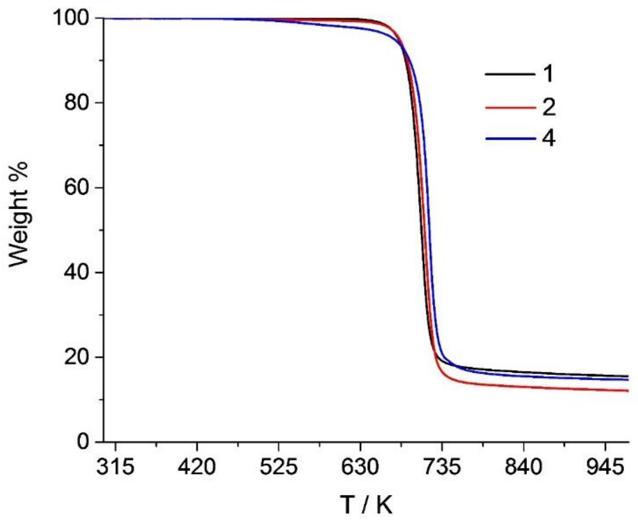

Supplementary Figure 20. Thermal stability of liquids. TGA profiles of compounds **1**, **2** and **4**. The 2% weight loss of compounds **1**, **2** and **4** were observed at 665, 664 and 606 K, respectively. A weight loss of 5% were found at 678, 680 and 673 K for compounds **1**, **2** and **4**, respectively.

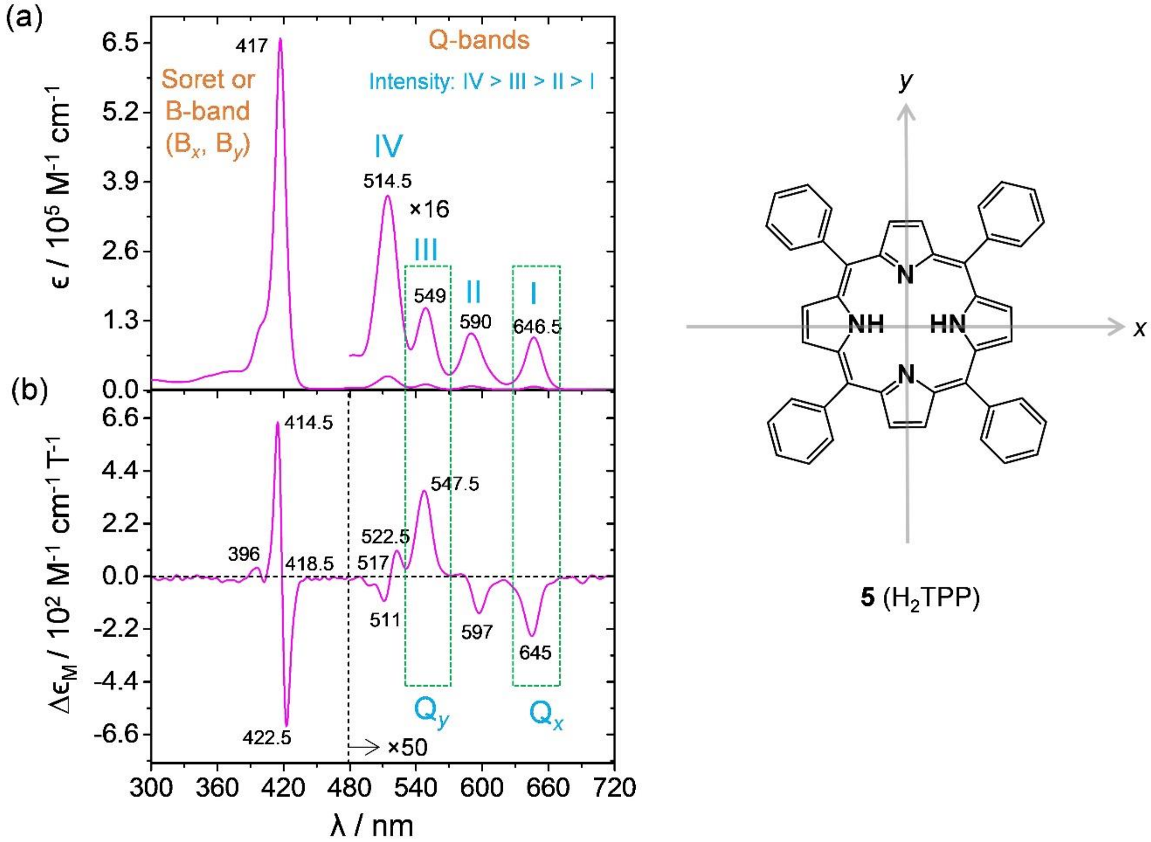

Supplementary Figure 21. Analysis of absorption and MCD signals of H_2TPP. UV-visible absorption (a) and the corresponding MCD (b) spectra of H_2TPP (compound **5**) recorded in dichloromethane. The concentrations of solution were 1.2×10^{-6} and 2×10^{-5} M respectively, for Soret and Q-band measurements. The arrows *x* and *y* represent the respective x-axis and y-axis of compound **5**.

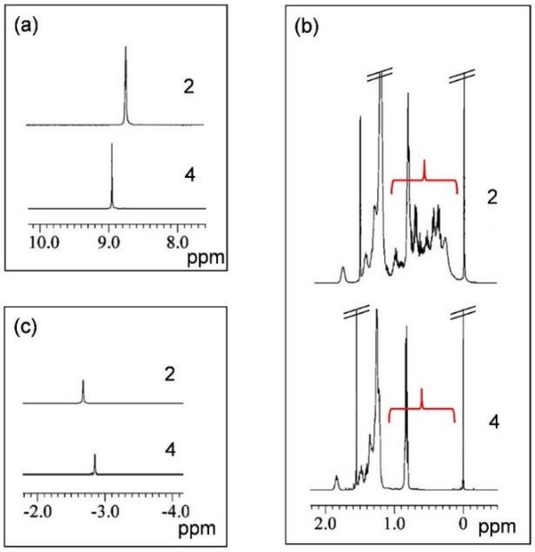

Supplementary Figure 22. Analysis of ^{1}H NMR spectra in solution. Comparison* of ^{1}H NMR spectra of compounds **2** and **4** in $CDCl_3$; (a) *β*-pyrrole protons, (b) alkyl chain protons and (c) inner NH protons.

* Contrast to compound **4**, little broad and multiplet proton signals in compound **2** were observed because of the possible atropisomers due to the alkyl-substitution at 2-position of *meso*-phenyl groups. The *β*-pyrrole protons of compound **2** were appeared at 8.74–8.75 ppm as multiplet, while the same were shifted downfield and appeared as singlet at 8.95 ppm (Supplementary Figure 22a) in compound **4**. Again, the alkyl protons signal of compound **2** were found to be more upfield shifted because of ring-current effect of porphyrin ring (Supplementary Figure 22b) which cover the protons of alkyl chains at the 2-position of *meso*-phenyls. The inner NH protons of porphyrin ring in compound **2** appeared at −2.68 ppm while in case of compound **4** it was shifted toward upfield and appeared at −2.83 ppm (Supplementary Figure 22c). Similarly, the $–OCH_2–$ protons of alkyl chains were appeared as two broad multiplets at 3.86–3.90 and 3.57–3.68 ppm in compound **2**, while in compound **4**, the same protons were shifted downfield and appeared as sharp doublet at 3.97 ppm (Supplementary Figures 4 and 8). These negligible shifts in proton signals clearly hints a distinct inequality in the electronic structures of compounds **2** and **4**. Noted, the ^{1}H NMR spectral pattern and chemical shift values were almost identical for the series of compounds **1–3** (Supplementary Figures 2,4,6,8).

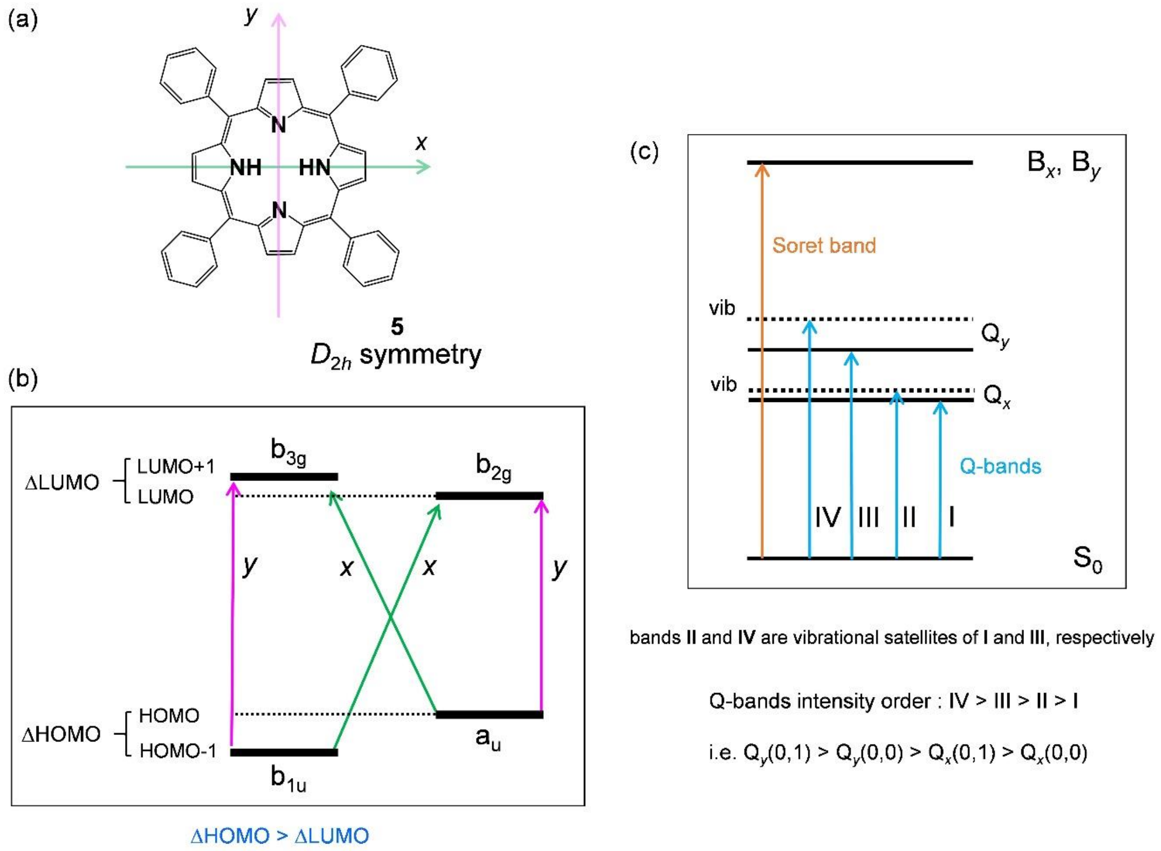

Supplementary Figure 23. Pictorial presentation of simplified Gouterman's "Four-Orbital" model. (a) The compound **5** possess a D_{2h} symmetry. (b) Schematic illustration showing relative value of ΔHOMO and ΔLUMO as well as possible interactions among the four frontier molecular orbitals. (c) A simplified diagram representing the Soret and Q-band transitions and their relative intensity order in compound **5**.

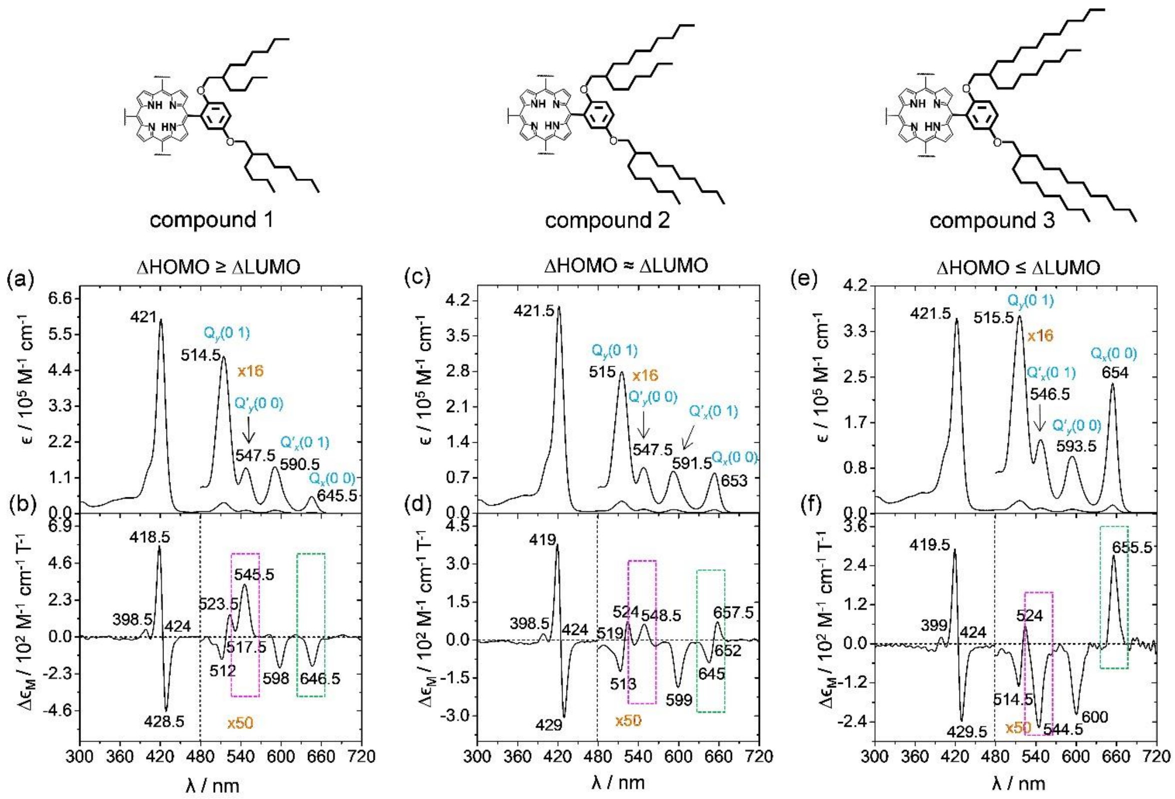

Supplementary Figure 24. Assignments of Q-band transitions. UV-visible absorption (a, c and e) and the corresponding MCD (b, d and f) spectra of compounds **1**, **2** and **3** respectively, recorded in dichloromethane. The energy order of Q-bands in compound **5** follow the trend $Q_x(0,0) < Q_x(0,1) < Q_y(0,0) < Q_y(0,1)$ (Supplementary Figure 23). Similarly, compounds **1–3** also showed the identical trend of Q-band energy order. However, a large difference between UV-visible absorption peak maxima and their corresponding MCD peak maxima was observed mainly for the vibrational satellites $Q_x(0,1)$ and $Q_y(0,1)$ which are marked as $Q'_x(0,1)$ and $Q'_y(0,1)$. These results suggested a probable mixing of these vibrational satellites with the next vibrational components or adjacent Q/Soret components.

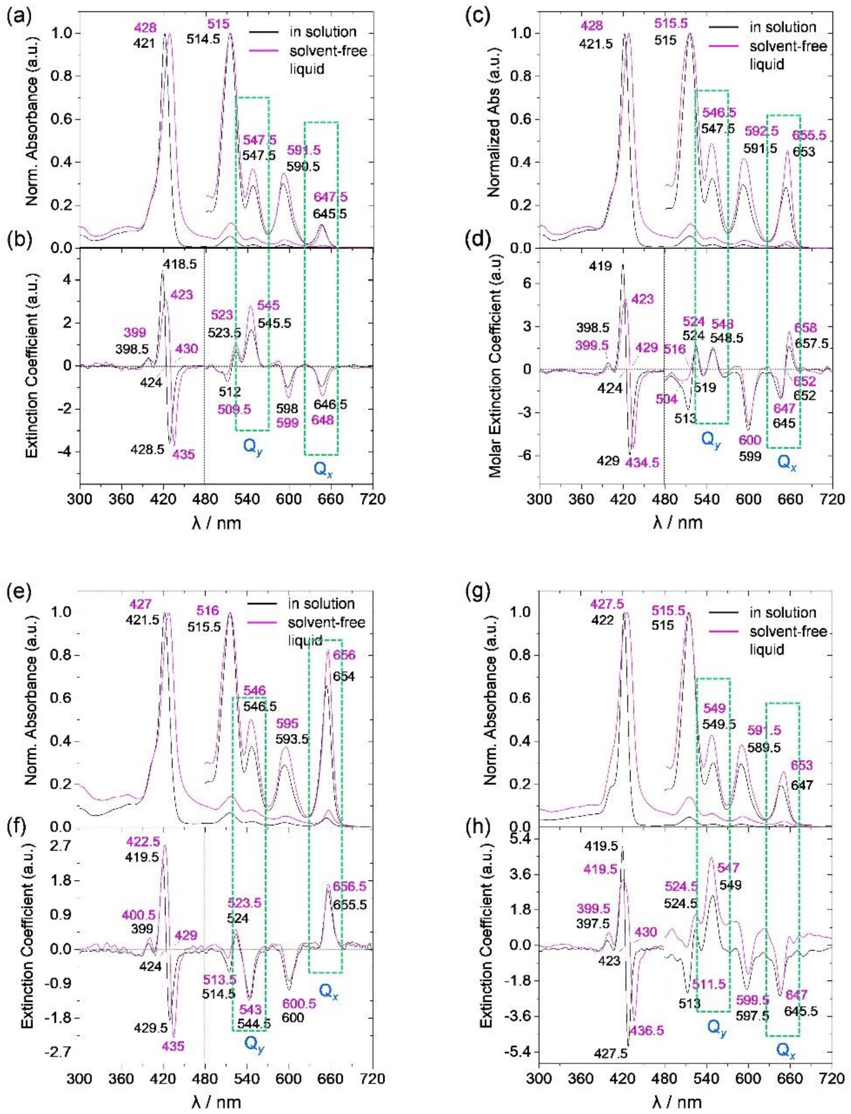

Supplementary Figure 25. Molecular intrinsic properties in condensed liquid state. Comparison of solution (dichloromethane) and solvent-free liquid state UV-visible absorption (a, c, e and g) and the corresponding MCD spectra (b, d, f and h) of compounds **1–4**, respectively. The UV-vis absorption spectra were normalized. These spectral analyses reveal a similar spectral feature of solution and solvent-free liquid state in compounds **1–4**. Interestingly, the change in the sign sequences of MCD signals follow exactly similar trend under both conditions. Therefore, we conclude that the inherent optoelectronic and structural properties of these porphyrins were also retained in bulk liquid state.

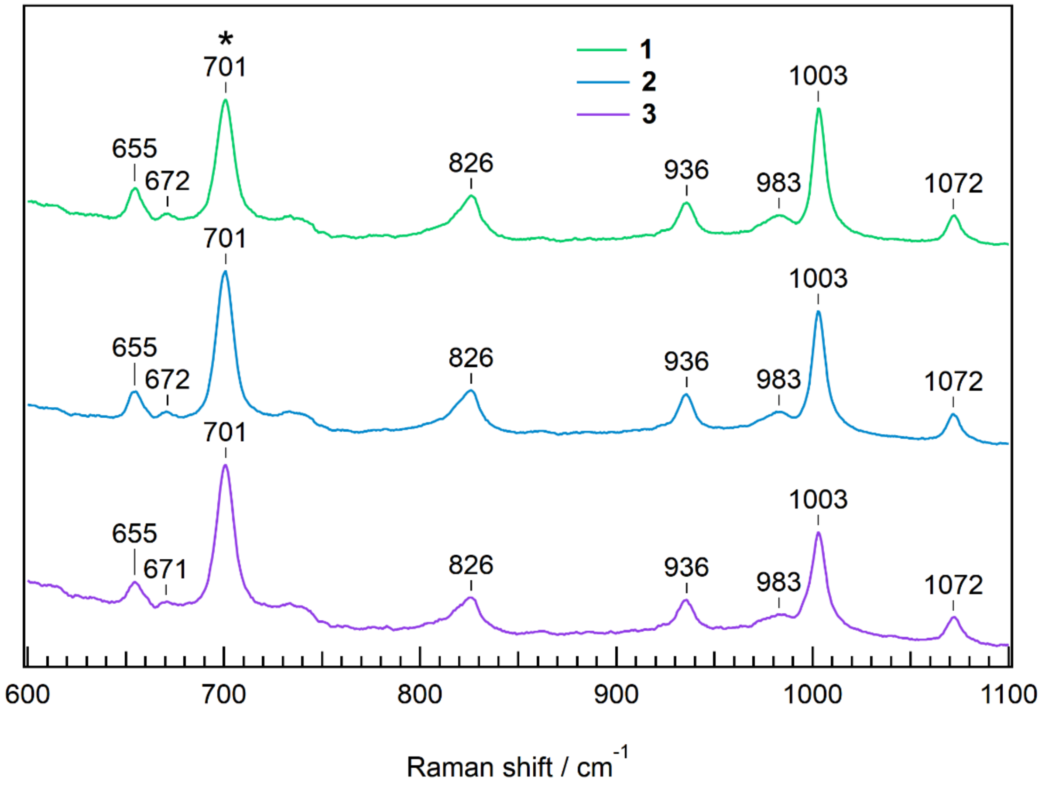

Supplementary Figure 26. Resonance Raman spectra in low frequency region. Comparison of resonance Raman (RR) spectra of compounds **1–3** in 600–1100 cm^{-1}. The spectra were recorded in CH_2Cl_2 solution of 1×10^{-5} M concentration. The band at 701 cm^{-1} is due to the solvent. The spectra in this frequency region are identical for compounds **1–3**.

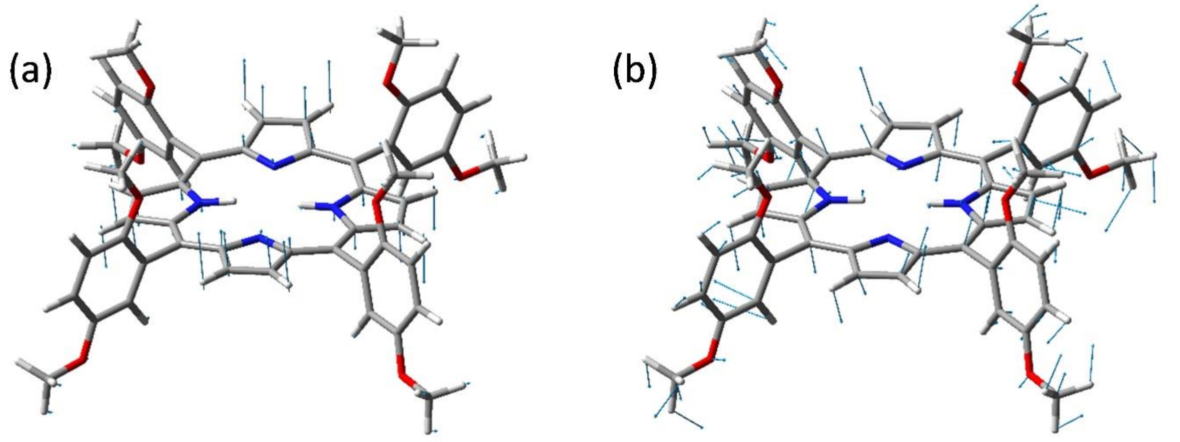

Supplementary Figure 27. Calculations of displacement vectors. (a) Displacement vectors characterized by the saddling distortion along with the normal modes with the frequency of 78 cm^{-1}. (b) Displacement vectors characterized by the ruffling distortion along with the normal modes with the frequency of 273 cm^{-1}.

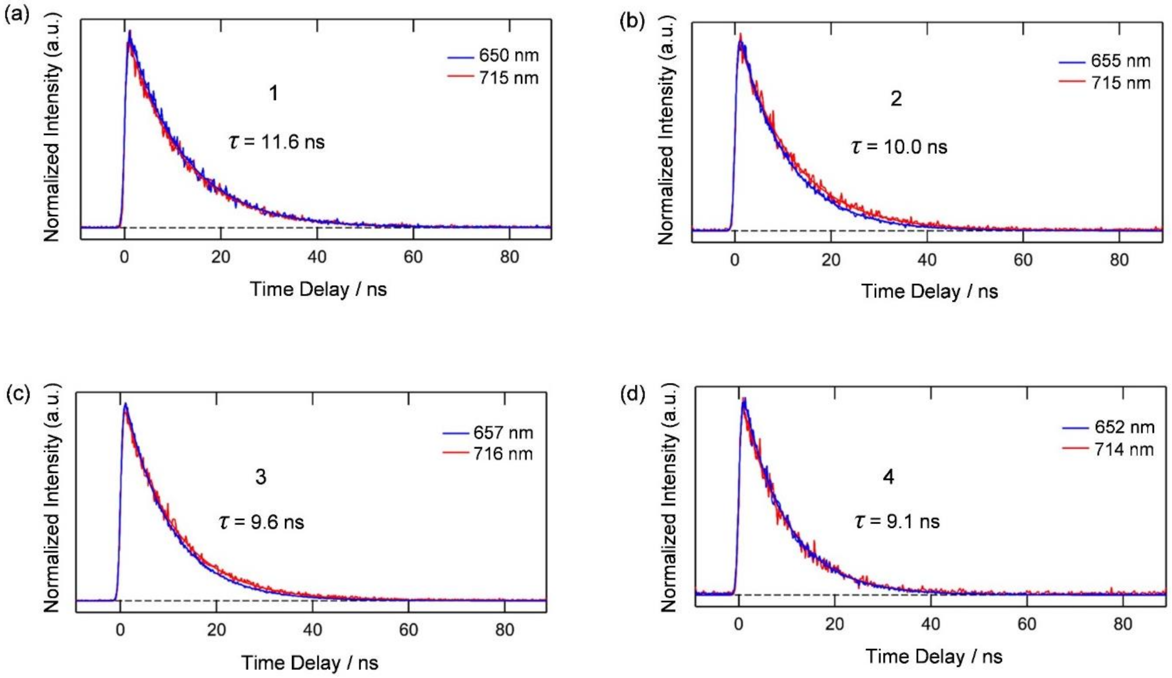

Supplementary Figure 28. Fluorescence decay profile. Fluorescence decay profiles of compounds **1–4** (a-d, respectively) obtained from time resolved fluorescence measurements in toluene. The data were monitored at their both emission maxima of the respective compounds.

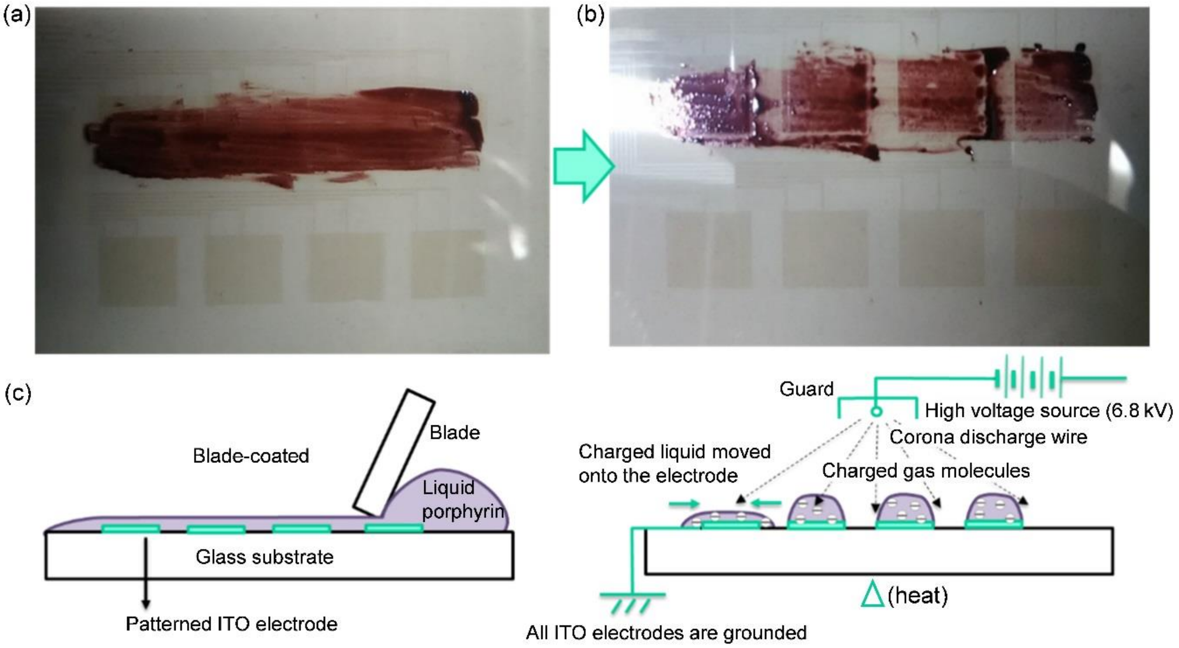

Supplementary Figure 29. Liquid electret device fabrication and corona charging process. Photographs of (a) before and (b) after corona charging of the liquid-porphyrin coated on patterned ITO glass electrode. (c) Schematic diagram of the fabrication process of liquid-electret device. During the process of corona discharge, the liquid-porphyrin slowly moved onto the bottom electrodes connected to the ground (b), which indicating their charged state.

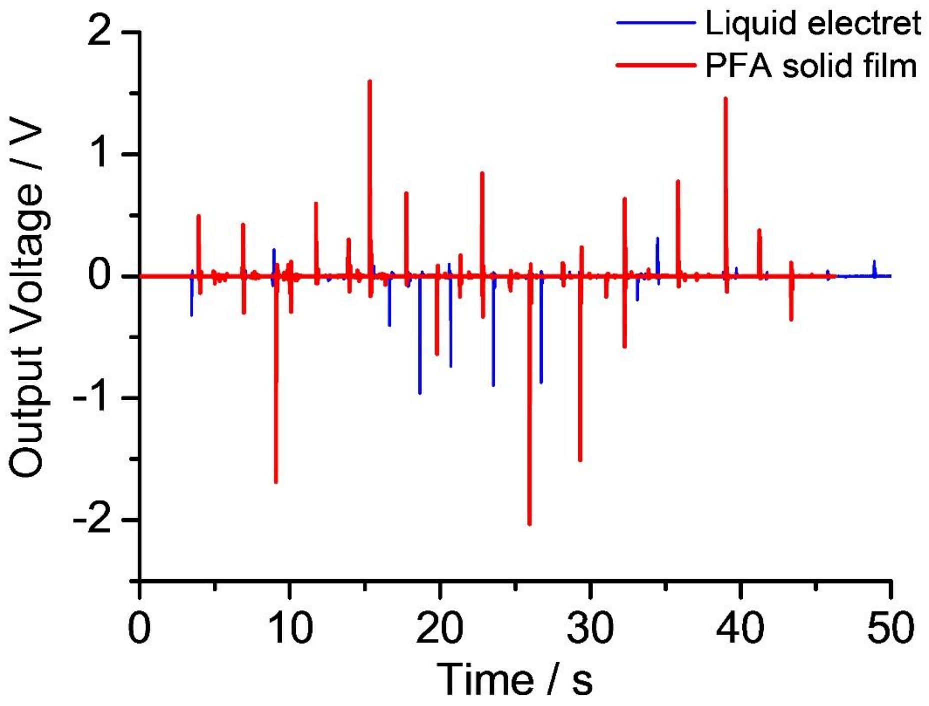

Supplementary Figure 30. Output voltage characteristics between solid and liquid electret devices. Comparison of output voltage characteristics between conventional solid film-type electret composed of copolymer of tetrafluoroethylene and perfluoroalkoxyethylene (PFA) and liquid-electret devices. The output voltage measurements were conducted with fleshly prepared samples after corona charging (within 12 h, as described in the stability test; Fig. 5e).

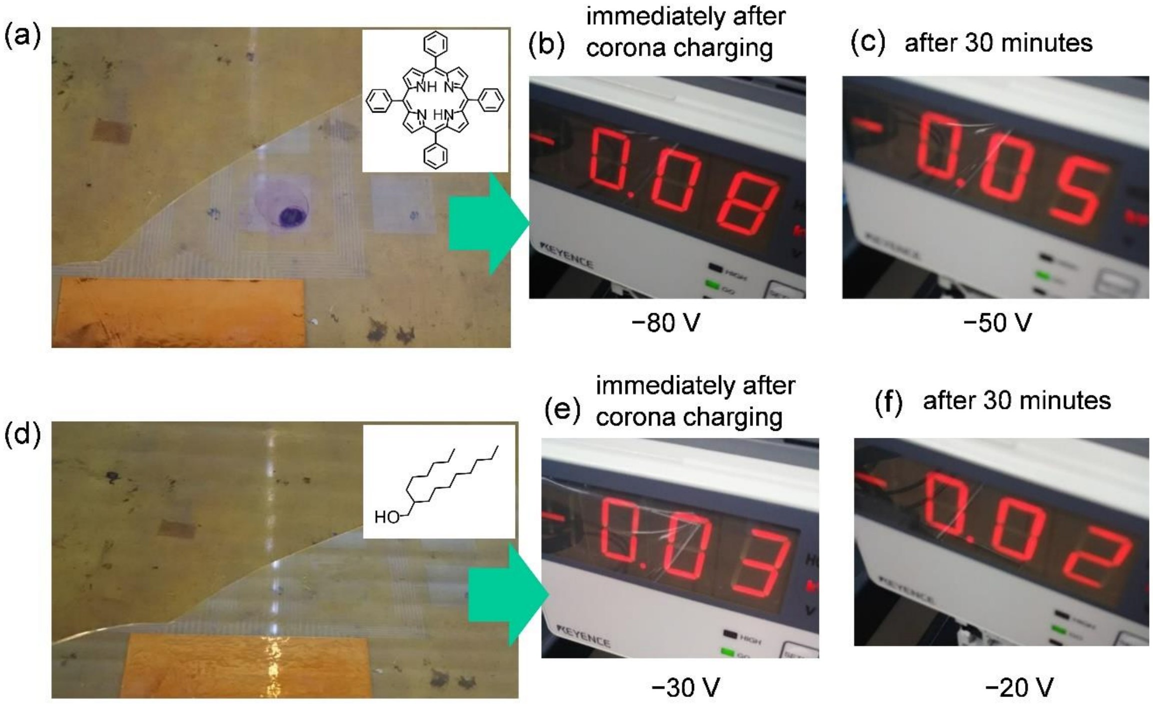

Supplementary Figure 31. Control experiments of corona charging. (a) Photograph of the device fabricated with solid H_2TPP (compound **5**). Display of output voltages of the device with compound **5** (b; immediately after corona charging) and (c; 30 min after corona charging). (d) Photograph of the device fabricated with pure liquid alkyl chain, 2-hexyl-1-decanol. Display of output voltages of 2-hexyl-1-decanol (e; immediately after corona charging) and (f; 30 min after corona charging). Unlike liquid porphyrin (Supplementary Figure 29b), the compound **5** and 2-hexyl-1-decanol were not moved onto the electrode during the process of corona discharge.

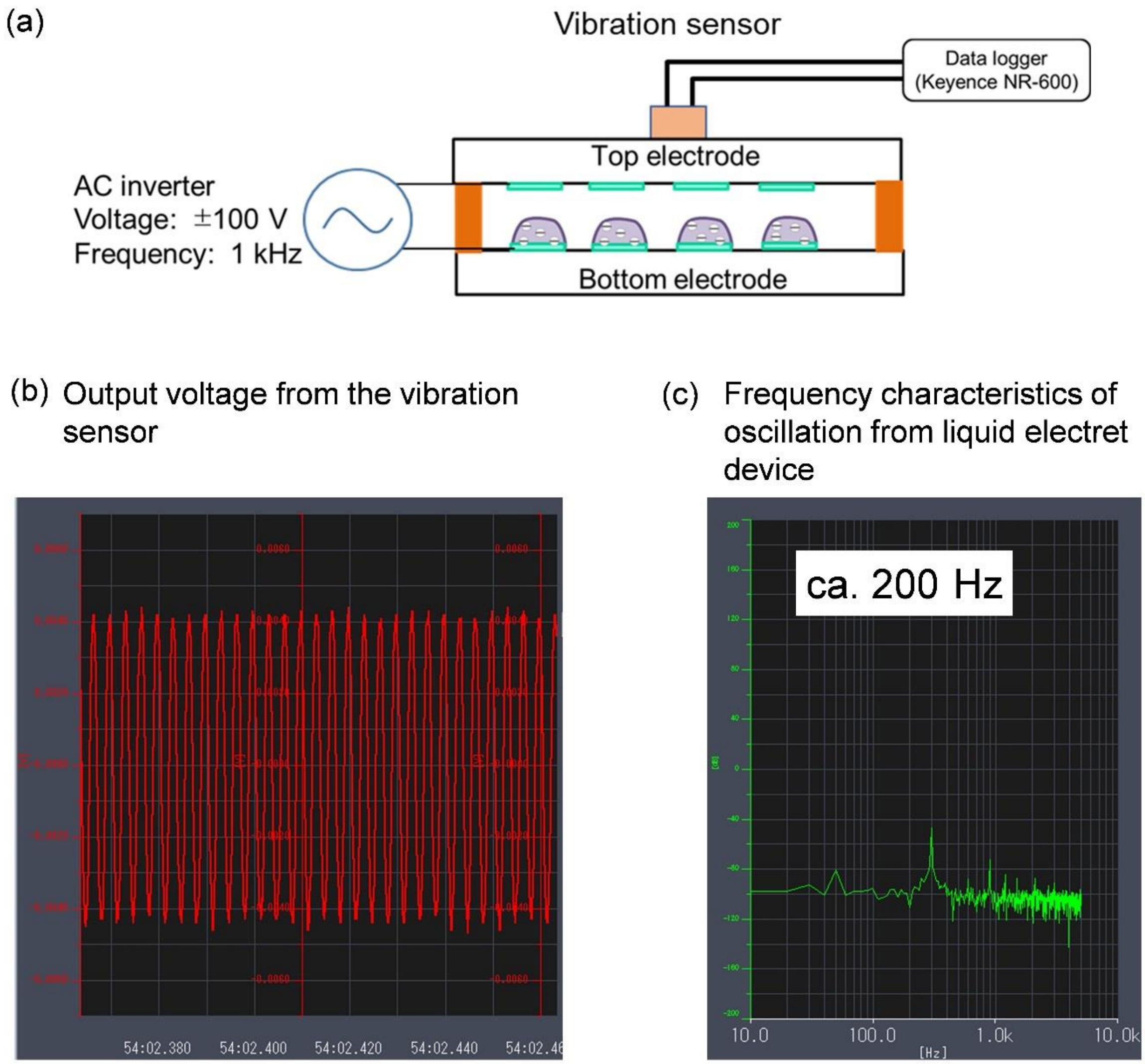

Supplementary Figure 32. Electroacoustic performance of liquid electret. Schematic diagram of the cross-sectional view of the liquid-electret based electroacoustic device (a). Oscillation output characteristics of the liquid-electret electroacoustic device (b) and (c). In the oscillation characteristics measurement (c), an output of oscillation frequency ca. 200 Hz was obtained by applying 1 kHz input frequency from the AC inverter. The viscoelastic properties of the liquid-electret could be the reason for such downshift of output frequency.

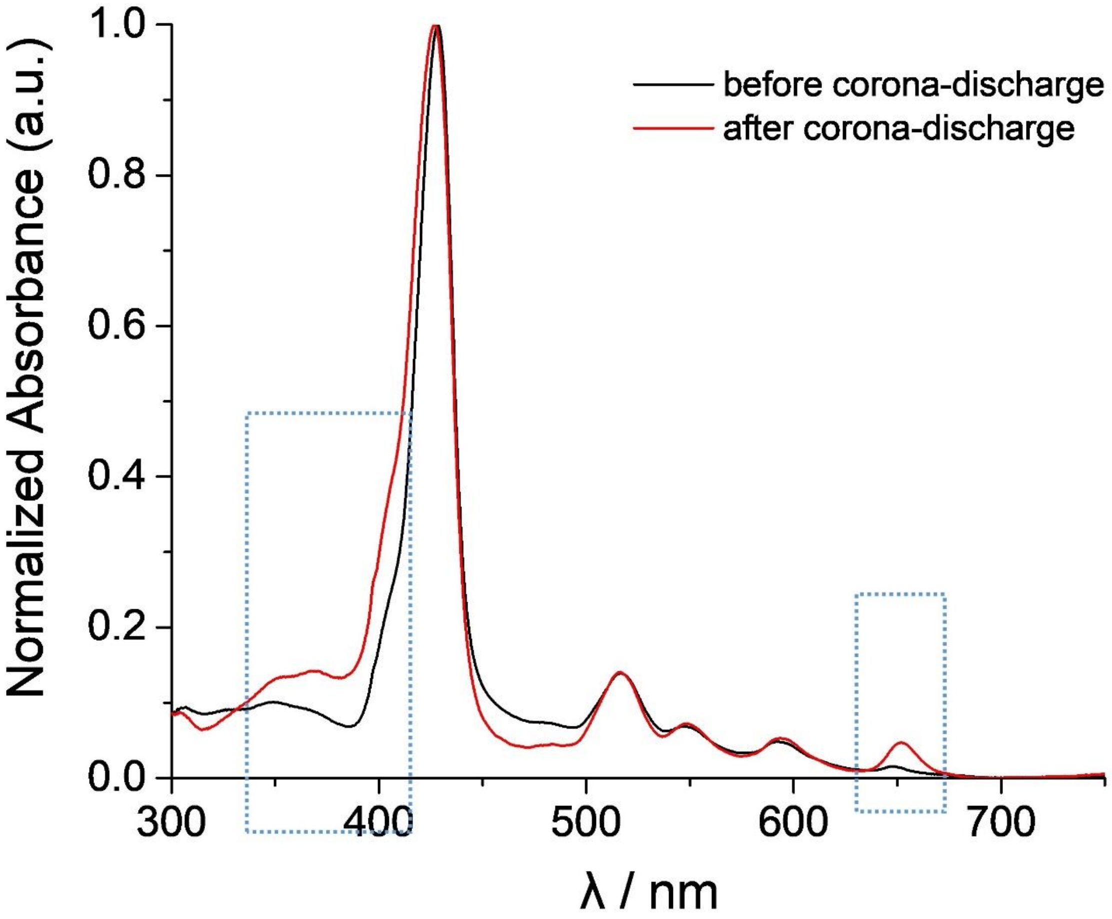

Supplementary Figure 33. Electronic effects on corona charging process. Comparison of absorption spectra of a film of compound **2**; before and after corona-discharge process. For this experiment, a thin layer of compound **2** was prepared on the conducting surface of ITO glass.

Supplementary Tables

Supplementary Table 1. Data of UV-visible absorption, emission, glass transition, viscosity and sign variation in MCD of compounds **1–5**.

compounds	UV-vis absorption[a]				fluorescence[a]	glass transition temperature[b] T_g (K)	complex viscosity[c] η^* (Pa·s)	MCD sign sequence		
	in CH_2Cl_2 λ_{max} (ε / 10^4 M^{-1} cm^{-1})		solvent-free liquid state, λ_{max}		in toluene λ_{em} (0–0)					
	Soret-band	Q-bands	Soret-band	Q-bands				B[d]	Q_y	Q_x
**1**	421<br>(59.72)	514.5 (3.02)<br>547.5 (0.88)<br>590.5 (0.90)<br>645.5 (0.33)	428	515<br>547.5<br>591.5<br>647.5	649	247	106.9	+/-	+	-
**2**	421.5<br>(40.87)	515 (2.32)<br>547.5 (0.75)<br>591.5 (0.68)<br>653 (0.66)	428	515.5<br>546.5<br>592.5<br>655.5	655	228	15.7	+/-	+	-/+
**3**	421.5<br>(35.71)	515.5 (2.26)<br>546.5 (0.84)<br>593.5 (0.65)<br>654 (1.49)	427	516<br>546<br>595<br>656	656	212	–	+/-	-	+
**4**	422<br>(63.73)	515 (2.70)<br>549.5 (0.81)<br>589.5 (0.79)<br>647 (0.52)	427.5	515.5<br>549<br>591.5<br>653	652	225	75.1	+/-	+	-
**5**	417<br>(65.87)	514.5 (2.27)<br>549 (0.96)<br>590 (0.66)<br>646.5 (0.61)	–	–	–	–	–	+/-	+	-

[a] λ are in nm. [b] Offset temperature (as the T_g are distributed in a wide temperature range). [c] At angular frequency (ω) = 25.1 rad s^{-1} and T = 298 K. [d] Soret band B (B_x, B_y). Q_x and Q_y have been defined in Fig. 3c,d and Supplementary Figure 21.

Supplementary Table 2. Comparison of observed and calculated vibrational frequencies of compounds **1–3**.

observed frequency / cm^{-1}			calculated frequency / cm^{-1}	symmetry species	mode
**1**	**2**	**3**			
1586	1585	1586	1595	A_{1g}	ϕ_4
1554	1555	1556	1574	A_{1g}	ν_2
—	**1541**	**1542**	**1545**	$\mathbf{E_u}$	$\boldsymbol{\nu_{37}}$
—	**1522**	**1523**	**1535**	$\mathbf{E_u}$	$\boldsymbol{\nu_{38}}$
1497	1497	1497	1520	B_{1g}	ν_{11}
1446	1446	1445	1460	A_{1g}	ν_3
1382	1382	1382	1409	B_{2g}	ν_{29}
1360	1360	1359	1372	A_{1g}	ν_4
1324	1324	1324	1365	A_{2g}	ν_{20}
1291	1291	1291	1309	B_{1g}	ν_{12}
—	**1204**	**1204**	**1248**	$\mathbf{E_u}$	$\boldsymbol{\nu_{51}}$
1180	1180	1180	1213	A_{1g}	ν_1
1123	1223	1122	1126	A_{1g}	ϕ_6

The RR frequencies calculated by considering D_{2d} symmetry were found to match closely with the experimentally obtained vibrations. The three highlighted E_u modes were identified as the three newly-appeared RR bands in compounds **2** and **3** (Fig. 4a). For detail analysis of RR spectra see the following section 'assignments of RR bands'.

Supplementary Table 3. Correlation table for the species of D_{4h} group and its subgroups (D_{2h}, D_{2d} and S_4).

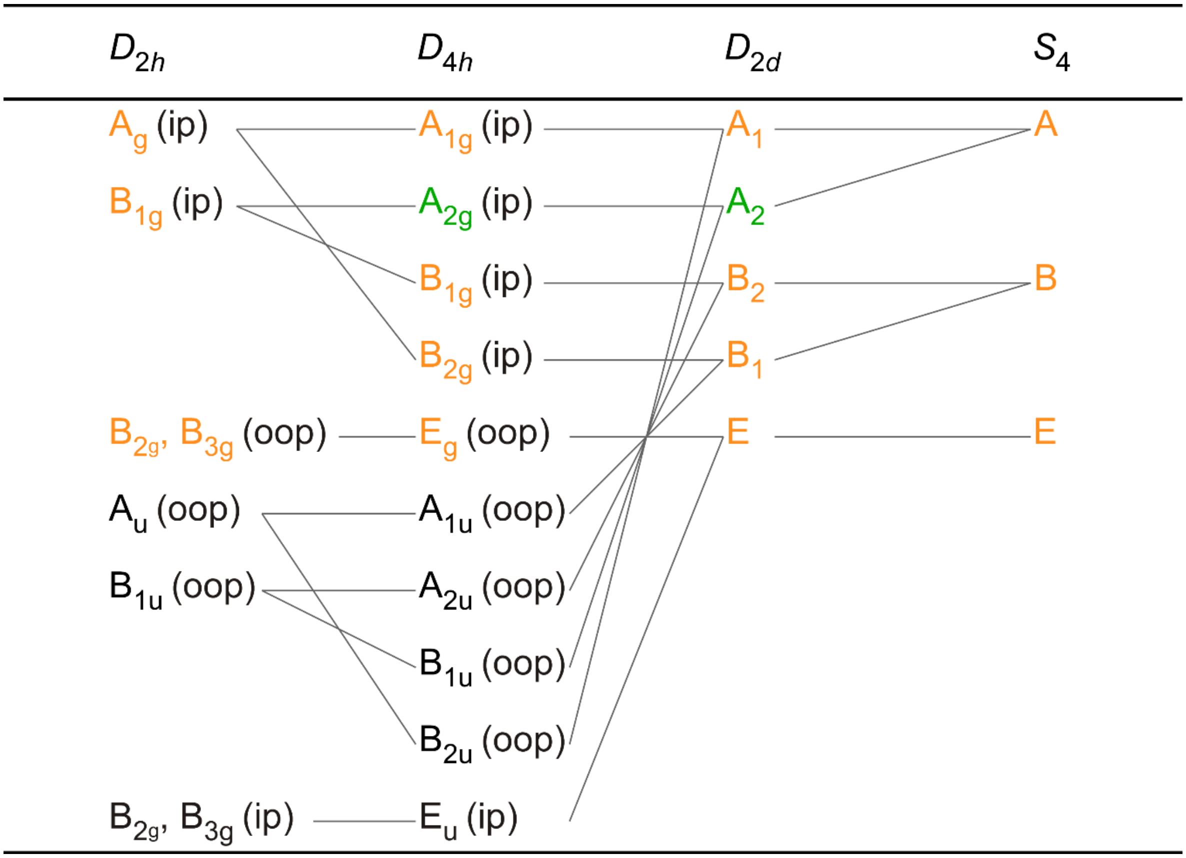

Orange colour: Raman-active; green colour: Raman-active in resonance condition
ip: in-plane mode and oop: out-of-plane mode

Supplementary Table 4. Calculated energy gaps of compound **6**, i.e. ΔHOMO=$E_{(HOMO)}$–$E_{(HOMO-1)}$ and ΔLUMO=$E_{(LUMO+1)}$–$E_{(LUMO)}$, at the equilibrium structure and the displaced geometries along the saddling and ruffling distortion. The norm of the displacement vectors is shown in the parenthesis.

Orbital energy gap	Equilibrium (cm^{-1})	Saddling(|δ|=2Å) (cm^{-1})	Ruffling(|δ|=2Å) (cm^{-1})
ΔLUMO	287	243	875
ΔHOMO	1272	702	2600

Supplementary Notes

Supplementary Note 1: MCD spectral analysis. Gouterman's "Four-Orbital" model for free-base porphyrin of D_{2h} symmetry (Supplementary Figure 23) suggested that the changes in intensity and energy of absorption spectra would be associated with the difference of two highest occupied molecular orbitals (HOMO and HOMO–1; i.e. ΔHOMO) and two lowest unoccupied molecular orbitals (LUMO and LUMO+1; i.e. ΔLUMO). The values of ΔHOMO and ΔLUMO, which are often small in the case of tetraphenylporphyrin derivatives, can distinctly influence the absorption spectra. On the other hand, the relative magnitude of ΔHOMO and ΔLUMO is very sensitive towards any electronic influences to the porphyrin-core. The anomaly observed in the Q-band intensity order of absorption spectra in compounds **1**–**3** (Fig. 3c) are associated with the relative magnitude in the ΔHOMO and ΔLUMO values.

Therefore, the magnetic circular dichroism (MCD) studies which provide a better picture of ground and excited state degeneracy arising from the Zeeman splitting of electronic states under an external magnetic field,[6–8] were further performed to detect the nature of ΔHOMO and ΔLUMO.

The MCD spectra of compounds **1**–**3** (Fig. 3d and Supplementary Figure 24) in dichloromethane exhibited very similar derivative-shaped signals corresponding to the Soret bands, which were identified as pseudo-Faraday *A* term (for D_{2h} symmetry molecule, no degenerate state exists). Figure 3d clearly indicated that the negligible changes in the UV-visible absorption spectra particularly at Q-bands **I** and **III** (Q_x and Q_y, respectively) appeared as distinctly different signals in the corresponding MCD spectra. For instance, with increasing alkyl chain lengths in compounds **1**–**3**, the sign of Q_x signal (identified as Faraday *B* term) gradually changed to a completely opposite sign. For example, the band-**I** appeared as a negative trough in compound **1**, while in compound **3** it appeared as a positive peak although in compound **2** it appeared as an intermediate showing both positive and negative envelopes. A similar reversal of sign sequence was also observed for band-**III**. It should be noted that in all cases the sign sequence of B-band remains unchanged. This kind of unique reversal of sign sequence in MCD spectra is quite unusual.[9–11] Now, the relative values of

ΔHOMO and ΔLUMO can be predicted from the sign sequence of MCD signals of the Q and B (B_x, B_y; Soret) bands.[S6] In case of conventional free-base porphyrin **5**, which has D_{2h} symmetry (or not higher than C_3 symmetry), the ΔHOMO > ΔLUMO relationship generally exists (Supplementary Figure 23b). Namely in compound **5**, the sign of MCD signals for Q_x, Q_y and B (B_x, B_y) bands follow the sequence –, +, –/+ respectively, in ascending energy (Supplementary Figure 21b and Supplementary Table 1). Comparison of this result with Figure 3d revealed that only compound **1** follows the similar sign sequence as that of compound **5**. Therefore, we considered that the similar ΔHOMO > ΔLUMO relationship exists for compound **1**. According to Michl's demonstration[12] the change in sign of Q MCD signals can be explained by considering "soft MCD chromophores" in which ΔHOMO ≈ ΔLUMO. In these chromophores, two extreme situations ΔHOMO > ΔLUMO or ΔLUMO > ΔHOMO can occur even by a small alteration in the induced excited-state magnetic moments. In contrast to compound **1**, therefore, the complete reversal of Q MCD sign in compound **3** appears possible if ΔLUMO becomes larger than ΔHOMO. Accordingly, we postulated that with increasing chain lengths in compounds **1**–**3**, the relative values of ΔLUMO and ΔHOMO varied by the following sequence ΔHOMO ≥ ΔLUMO (compound **1**), ΔHOMO ≈ ΔLUMO (compound **2**) and ΔHOMO ≤ ΔLUMO (compound **3**), respectively (Supplementary Figure 24).

In the main text, we have discussed that the subtle structural distortion in the porphyrin ring was the main reason for such perturbation in ΔHOMO and ΔLUMO values. Although the effect is not directly related, this type of structural perturbation that disrupts the orbital angular momentum (OAM) properties of the LUMOs to a greater extent than those of HOMOs, was reported for zinc tetraphenyltetraacenaphthoporphyrin with a saddling distortion.[8]

Now the fully electric dipole forbidden $Q_x(0,0)$ transition gains intensity only through vibrational borrowing from the allowed B(0,0) band which resulted in concomitant decrease in Soret-band intensity (Fig. 3d) of compounds **1**–**3**.

Supplementary Note 2: Assignments of RR bands. To assign the vibrational bands of compounds **1**–**3**, an effective D_{4h} symmetry was applied. In D_{4h} symmetry, in-plane vibrational modes are classified to five symmetries; A_{1g}, A_{2g}, B_{1g}, B_{2g}, and E_u. The modes except for E_u mode are active in RR spectra. Vibrational assignments are summarized in Supplementary Table 2. In resonance with the B band, the RR spectrum of compound **1** is dominated by bands arising from totally symmetric modes, A_{1g} modes. In addition to the A_{1g} modes, B_{1g} and phenyl substituent modes were observed in the spectra.

It should be noted that new bands appeared at 1522–1523 and 1541–1542 cm^{-1} in compounds **2** and **3** and became prominent as the alkyl chains became longer while the bands are absent in compound **1**. These bands cannot be assigned to in-plane modes of the porphyrin by the following reasons. In 1400–1600 cm^{-1}, in-plane vibrational modes due to stretching modes of the C_β–C_β and C_α–C_m bonds are observed. They are ν_2 (A_{1g}), ν_3 (A_{1g}), ν_{10} (B_{1g}), ν_{11} (B_{1g}), ν_{28} (B_{2g}), and ν_{19} (A_{2g}) modes. The possibilities of the assignments to ν_2, ν_3, or ν_{11} modes can be ruled out because bands due to these modes were observed at 1554–1556, 1445–1446, and 1497 cm^{-1}, respectively. The frequencies of ν_{10} and ν_{28} modes for free-base TPP are 1574 and 1485 cm^{-1},[13] which are much different from the observed frequencies. Bands due to A_{2g} modes are not usually observed in RR spectra with the B band excitation because they are not Jahn-Teller active. Accordingly, the bands at 1522–1523 and 1541–1542 cm^{-1} cannot be assigned to any in-plane modes in D_{4h} symmetry.

The increase of the observed vibrational modes strongly suggests lowering of symmetry of the porphyrin macrocycle. Supplementary Table 3 describes correlations of species of D_{4h} group and those of its subgroups. Symmetry lowering to planar D_{2h} symmetry does not result in the increase of Raman or RR active modes. Thus, the appearance of the bands at 1523 and 1542 cm^{-1} indicates nonplanarity of porphyrin macrocycles in compounds **2** and **3**. In D_{2d} (saddled) and S_4 (ruffled) symmetry, the vibrational modes of A_{1u}, A_{2u}, B_{1u}, B_{2u}, and E_u in D_{4h} symmetry become Raman or RR active in addition to A_{1g}, A_{2g}, B_{1g}, B_{2g}, and E_g modes. A_{1u}, A_{2u}, B_{1u}, and B_{2u} modes are out-of-plane vibrations while E_u mode is an

in-plane vibration. The bands at 1522-1523 and 1541-1542 cm^{-1} should be assigned to E_u modes because out-of-plane modes are expected at low frequencies, 1100 cm^{-1}.[14]

Supplementary Methods

Chemical. 2-Butyl-1-octanol, 2-hexyl-1-decanol, 2-octyl-1-dodecanol, pyrrole, a part of 2,5-dihydroxy benzaldehyde and 3,5-dihydroxy benzaldehyde were purchased form Sigma-Aldrich. Propionic acid, trifluoroacetic acid, 5,10,15,20-tetraphenylporphyrin (H_2TPP), 5,10,15,20-tetrakis-(3,5-dimethoxyphenyl)porphyrin, a part of 2,5-dihydroxy benzaldehyde and *3,5*-dihydroxy benzaldehyde were obtained from TCI chemicals. Carbon-tetrabromide, triphenylphosphine (PPh_3), zinc acetate dihydrate [$Zn(OAc)_2.2H_2O$], potassium carbonate (K_2CO_3), potassium iodide, spectroscopic grade solvents like dichloromethane, toluene, and reaction grade ethanol were purchased from Wako chemical. Sodium sulphate (Na_2SO_4), triethyl amine (NEt_3), dehydrated THF and DMF, NMR-solvent $CDCl_3$, common solvents for chromatographic purification such as dichloromethane, *n*-hexane, chloroform, toluene and silica gel 60 N (spherical, neutral) of 40–50 µm were supplied by Kanto Chemical. Bio-Beads S-X1-support, 200–400 mesh was purchased form Bio-Rad Laboratories. All chemicals and solvents were used as received.

Instrumentation. The ^{1}H and ^{13}C NMR (δ in parts per million) spectra in $CDCl_3$ were recorded in a JEOL ECS-400 MHz spectrometer of frequency 400 and 100 MHz respectively. Tetramethylsilane (TMS) was used as an internal reference for recording ^{1}H NMR spectra in $CDCl_3$ (residual proton; δ = 7.24 ppm). Solid state NMR data for solvent-free liquid samples were collected in Bruker Avance III 600 MHz spectrometer. Solid state ^{1}H and ^{13}C NMR spectra were collected under magic angle spinning (MAS) conditions of 13.5 kHz and 12.5 kHz respectively. In ^{13}C MNR, the chemical shifts were adjusted to 176.03 ppm of glycine carbonyl carbon with reference to tetramethylsilane (0.0 ppm). The cross polarization-magic angle spinning (CP-MAS) and direct single pulse excitation with dipolar decoupling-magic angle spinning (DD-MAS) methods were used for ^{13}C NMR spectra. Generally, 10000

transients were accumulated and 10 Hz of Lorentzian line broadening function was applied prior to Fourier transformation. The spinning frequency was fixed to 12.5 kHz and the duration of 90° pulse of observed ^{13}C nucleus was 5.0 us. In both CP-MAS and DD-MAS modes, two-pulses phase-modulation (TPPM) proton decoupling[1] was used with the contact and repetition times of 1 ms and 4 s, respectively. The solvent-free liquid sample (~30–40 mg) was placed into a zirconia rotor of 4.0 mm outer diameter and was capped tightly with a Teflon spacer and Kel-F cap.

The matrix-assisted laser desorption ionization time-of-flight (MALDI-TOF) mass spectra were recorded with a Shimadzu AXIMA-confidence mass spectrometer. UV-vis absorption spectra were obtained with JASCO-V670 spectrophotometer. Magnetic circular dichroism (MCD) spectra were recorded on a JASCO J-720 spectropolarimeter equipped with a JASCO MCD-104 electromagnet, which is capable to generate magnetic fields of up to 0.8 T. A constant field of a magnitude of 0.65 T was applied to sample solutions during the measurements. Optical microscopy images under polarized and cross-polarized light were obtained by using an Olympus BX51 optical microscopy system. The flash-photolysis time resolved microwave conductivity (FP-TRMC) experiment was performed by using a X-band (9 GHz) microwave circuit at low power and a nanosecond laser irradiation at 355 nm with photon density of 9.1 x 10^{15} photons cm^{-2} $pulse^{-1}$. Solvent-free liquid samples were directly filmed on a quartz plate and was used for this measurement. The rheology experiments were carried out using an Anton Paar Physica MCR301 rheometer, using the parallel plate geometry (with diameter 25 mm) and a measuring sample thickness of 0.25 mm. Before the measurements strain amplitude scans were performed for determining the linear-viscoelastic region. Small and wide angle X-ray scattering (SWAXS) data were collected by using an Anton Paar SAXSess mc^2 instrument. Thermogravimetric analysis (TGA) and differential scanning calorimetry (DSC) were performed with a Hitachi TG/DTA 6200 and Hitachi DSC7000X instruments respectively. Heating rate of TGA and heating-cooling rate of DSC were used as 10 K per minute under nitrogen gas flow. The absolute density (d) of the liquids were measured by using micromeritics AccuPyc II 1340 (Gas Pycnometer) instrument under helium gas at room temperature.

Picosecond time-resolved fluorescence spectra were recorded with a lab-built time-resolved fluorescence spectrometer. Details of the spectrometer have been published elsewhere.[2,3] Briefly, output of a femtosecond Ti:sapphire laser system (Coherent Micra-5/Legend-Elite USP, 800 nm, 30 fs, 1 kHz) was frequency-doubled and used as the pump pulse. Fluorescence from sample solution was introduced to a 30 cm spectrograph (Acton SP-2358) and detected with a streak camera (Hamamatsu Photonics C10627). A polarization analyzer was set at 54.7^{o} with respect to the pump polarization for avoiding time-dependent changes of fluorescence intensity due to rotational relaxation. The sample solution was held in a 1 cm quartz cuvette and rapidly stirred with a magnetic stirrer for avoiding accumulation of photodamage in an irradiated volume.

Synthesis. The precursor alkylated-aldehydes **1a–4a** (Supplementary Figure 1a) were synthesized by following literature report.[4] After complete purification, the pure aldehydes **1a**–**4a** were further used for the synthesis of targeted porphyrin derivatives (compounds **1**–**4**).

General synthesis for compounds 1–4. Compounds **1–4** were synthesized by following typical porphyrin condensation reaction between pyrrole and corresponding aldehydes in 1.2:1 mole ratio respectively, refluxing in propionic acid for 4–5 h (Supplementary Figure 1b) in air.[5] The progress of reactions were monitored by UV-vis absorption and thin-layer-chromatography (TLC) analysis. After the reaction completed, the propionic acid was distilled off under reduced pressure and the resulting viscus oil was extracted in $CHCl_3$. The crude products were passed through silica-column several times by using 10–20% CH_2Cl_2 in *n*-hexane as eluent. An impurity which showed blue spot under UV-light in TLC, was unable to remove through silica-column chromatography purification for large scale products. This impurity was successfully separated by gel permeation chromatography (GPC) in toluene. However, at this stage, a trace amount of another impurity which always moved together with desired product in TLC, was completely unable to separate by using any chromatographic techniques. Further zinc(II)-metallation of this mixture and subsequent

demetallation followed by chromatography purification in each step produced highly pure compounds **1–4**. In the zinc metalation reactions, the compounds were refluxed with 20 equivalent of $Zn(OAc)_2{\cdot}2H_2O$ in $CHCl_3$–EtOH (3:1) mixture for 8–10 h under argon. The completion of metallation reaction was judged by UV-vis absorption analysis. The crude products were completely purified through silica-column by using 15–25% CH_2Cl_2 in *n*-hexane. These highly pure Zn(II)-derivatives were further demetallated by using excess trifluoroacetic acid in CH_2Cl_2 at 273 K. After complete demetallation, the excess acid was neutralized by addition of few drops of trimethylamine to the reaction mixtures. The reaction mixture was washed thoroughly with deionized water and extracted in CH_2Cl_2 and dried over Na_2SO_4. After purification of these products through silica-column by using 9–14% CH_2Cl_2 in *n*-hexane yielded a highly pure dark-violet viscus oily liquids. Noted that the possibility of the presence of corresponding chlorine in these liquids were excluded as no signals were observed corresponds to the chlorine in ^{1}H NMR spectra.

Liquid compound 1: Dark violet viscus oil. Yield 12%. ^{1}H NMR (400 MHz, $CDCl_3$), δ = 8.73-8.76 (m, 8H, *β*-pyrrole H), 7.50-7.54 (m, 4H, Ar), 7.19-7.27 (m, 8H, Ar), 3.88-3.91 (m, 8H, $–OCH_2–$), 3.57-3.65 (m, 8H, $–OCH_2–$), 1.78-1.84 (br, 8H, –CH–), –0.09-1.47 (m, 176H, alkyl H), –2.67 (s, 2H, NH) ppm. ^{13}C (400 MHz, $CDCl_3$), δ = 153.75, 153.66, 153.59, 152.17, 152.04, 133.06, 132.95, 132.69, 132.61, 131.09, 122.78, 122.66, 122.54, 115.63, 115.56, 115.20, 114.82, 113.73, 113.31, 113.12, 113.03, 112.91, 72.40, 72.13, 71.95, 71.84, 38.36, 37.43, 37.36, 32.05, 31.58, 31.53, 31.49, 31.27, 30.54, 30.28, 29.91, 29.29, 29.16, 28.28, 27.05, 26.43, 26.26, 26.16, 23.27, 22.86, 22.52, 22.47, 22.40, 22.33, 22.29, 14.30, 14.11, 14.07, 13.97, 13.72, 13.61 ppm. UV-vis, λ_{max} nm (log ε), CH_2Cl_2: 421 (5.77), 514.5 (4.48), 547.5 (3.94), 590.5 (3.96), 645.5 (3.52). MALDI-TOF mass: *m/z* calculated for $C_{140}H_{222}N_4O_8$, 2087.71 $[M]^+$; Found: 2083.78 $[M–4]^+$.

Liquid compound 2: Dark violet viscus oil. Yield 10%.^{1}H NMR (400 MHz, $CDCl_3$), δ = 8.74-8.75 (m, 8H, *β*-pyrrole H), 7.49-7.53 (m, 4H, Ar), 7.18-7.26 (m, 8H, Ar), 3.86-3.90 (m, 8H, $–OCH_2–$), 3.57-3.68 (m, 8H, $–OCH_2–$), 1.79-1.84 (br, 8H, –CH–), 0.21-1.52 (m, 240H,

alkyl H), –2.68 (s, 2H, NH) ppm. ^{13}C (100 MHz, $CDCl_3$), δ = 153.78, 153.70, 153.54, 152.36, 152.26, 152.04, 133.21, 133.10, 133.01, 132.59, 122.95, 122.60, 122.34, 115.70, 115.54, 115.24, 115.08, 114.00, 113.65, 112.92, 72.61, 72.20, 71.98, 71.68, 38.41, 37.57, 37.43, 32.07, 31.86, 31.58, 30.57, 30.25, 29.91, 29.79, 29.53, 29.30, 29.05, 27.06, 26.25, 26.19, 26.15, 26.11, 23.02, 22.86, 22.71, 22.61, 22.48, 22.39, 22.32, 22.27, 22.20, 14.30, 14.10, 14.01, 13.96, 13.92, 13.85 ppm. UV-vis, λ_{max} nm (log ε), CH_2Cl_2: 421.5 (5.61), 515 (4.36), 547.5 (3.87), 591.5 (3.82), 653 (3.82). MALDI-TOF mass: *m/z* calculated for $C_{172}H_{286}N_4O_8$, 2536.21 $[M]^+$; Found: 2535.07 $[M–1]^+$.

Liquid compound 3: Dark violet viscus oil. Yield ~1–2%.^1H NMR (400 MHz, $CDCl_3$), δ = 8.74-8.75 (m, 8H, *β*-pyrrole H), 7.45-7.65 (m, 4H, Ar), 7.16-7.28 (m, 8H, Ar), 3.80-3.90 (m, 8H, $–OCH_2–$), 3.54-3.72 (m, 8H, $–OCH_2–$), 1.79-1.84 (br, 8H, –CH–), 0.12-1.52 (m, 304H, alkyl H), –2.68 (s, 2H, NH) ppm. ^{13}C (100 MHz, $CDCl_3$), δ = 153.72, 153.58, 152.28, 152.07, 133.19, 133.08, 132.64, 122.90, 122.60, 115.55, 115.36, 115.27, 115.12, 113.64, 113.02, 72.57, 72.22, 72.02, 71.79, 38.46, 37.42, 32.09, 31.58, 30.57, 30.27, 29.83, 29.53, 29.42, 29.26, 27.12, 22.86, 15.05, 14.29 ppm. UV-vis, λ_{max} nm (log ε), CH_2Cl_2: 421.5 (5.55), 515.5 (4.35), 546.5 (3.92), 593.5 (3.81), 654 (4.17). MALDI-TOF mass: m/z calculated for $C_{204}H_{350}N_4O_8$, 2984.71 $[M]^+$; Found: 2983.09 $[M–1]^+$.

Liquid compound 4: Dark violet viscus oil. Yield 20%. ^1H NMR (400 MHz, $CDCl_3$), δ = 8.95 (s, 8H, *β*-pyrrole H), 7.35 (d, *J* (H,H) = 2.14 Hz, 8H, Ar), 6.87-6.88 (m, 4H, Ar), 3.97 (d, *J* (H,H) = 4.25 Hz, 16H, $–OCH_2–$), 1.81-1.87 (br, 8H, –CH–), 1.22-1.35 (m, 192H, $–CH_2–$), 0.80-0.83 (m, 48H, $–CH_3$), –2.83 (s, 2H, NH) ppm. ^{13}C (100 MHz, $CDCl_3$), δ = 158.69, 144.00, 120.14, 114.36, 101.17, 71.43, 38.28, 32.06, 32.03, 31.58, 30.21, 29.88, 29.76, 29.50, 27.04, 22.85, 14.29 ppm. UV-vis, λ_{max} nm (log ε), CH_2Cl_2: 422 (5.80), 515 (4.43), 549.5 (3.91), 589.5 (3.90), 647 (3.72). MALDI-TOF mass: *m/z* calculated for $C_{172}H_{286}N_4O_8$, 2536.21 $[M]^+$; Found: 2535.04 $[M–1]^+$.

Supplementary References

1. Bennett, A. E., Rienstra, C. M., Auger, M., Lakshmi, K. V. & Griffin, R. G. Heteronuclear decoupling in rotating solids. *J. Chem. Phys.***103**, 6951–6958 (1995).
2. Lu, F. *et al*. A guide to design functional molecular liquids with tailorable properties using pyrene-fluorescence as a probe. *Sci. Rep.* **7**, 3416 (2017).
3. Lu, F. *et al*. Experimental and theoretical investigation of fluorescence solvatochromism of dialkoxyphenyl-pyrene molecules. *Phys. Chem. Chem. Phys.* **20**, 3258–3264 (2018).
4. Li, H. *et al*. Alkylated-C_{60} based soft materials: regulation of self-assembly and optoelectronic properties by chain branching. *J. Mater. Chem. C* **1**, 1943–1951 (2013).
5. Adler, A. D. *et al*. A simplified synthesis for *meso*-tetraphenylporphine. *J. Org. Chem.* **32,** 476–476 (1967).
6. Michl, J. Magnetic circular dichroism of cyclic π-electron systems. 1. Algebraic solution of the perimeter model for the *A* and *B* terms of high-symmetry systems with a $(4N + 2)$-electron [*n*]annulene perimeter. *J. Am. Chem. Soc.* **100**, 6801–6811 (1978).
7. Michl, J. Electronic structure of aromatic π-electron systems as reflected in their MCD spectra. *Pure Appl. Chem.* **52,** 1549–1563 (1980).
8. Mack, J., Asano, Y., Kobayashi, N. & Stillman, M. J. Application of MCD spectroscopy and TD−DFT to a highly non-planar porphyrinoid ring system. New insights on red-shifted porphyrinoid spectral bands. *J. Am. Chem. Soc.* **127**, 17697–17711 (2005).
9. Keegan, J. D. *et al*. Magnetic circular dichroism studies. 60. Substituent-induced sign variation in the magnetic circular dichroism spectra of reduced porphyrins. 1. Spectra and band assignments. *J. Am. Chem. Soc.* **104**, 4305−4317 (1982).
10. Keegan, J. D. *et al*. Magnetic circular dichroism studies. 61. Substituent-induced sign variation in the magnetic circular dichroism spectra of reduced porphyrins. 2. Perturbed molecular orbital analysis. *J. Am. Chem. Soc.* **104**, 4317−4329 (1982).

11. Djerassi, C. *et al*. Sign variation in the magnetic circular dichroism spectra of free-base porphyrins having a single π-acceptor pyrrole ring substituent. Structure implications[1]. *J. Am. Chem. Soc.* **106**, 4241−4258 (1984).
12. Michl, J. Magnetic circular dichroism of cyclic π-electron systems. 2. Algebraic solution of the perimeter model for the B terms of systems with a $(4N + 2)$-electron [*n*]annulene perimeter. *J. Am. Chem. Soc.* **100**, 6812–6818 (1978).
13. Saini, G. S. Resonance Raman study of free-base tetraphenylporphine and its dication. *Spectrochim. Acta, Part A* **64**, 981–986 (2006).
14. Spiro, T. G. & Li, X.-Y. Resonance Raman spectroscopy of metalloporphyrins. *In Biological Application of Raman Spectroscopy*. Spiro, T. G. Ed. Vol. III, p. 1–37 (John Wiley and Sons, New York, 1988).
