# Supplementary material for: Soft chromophore featured liquid porphyrins and their utilization toward liquid electret applications
Source: Nat Commun. 2019 Sep 30;10:4210. doi: 10.1038/s41467-019-12249-8 (PMC6768991; doi:10.1038/s41467-019-12249-8)
Supplement: Supplementary file 2 — Description of Additional Supplementary Files [file 41467_2019_12249_MOESM2_ESM.pdf]

Description of Additional Supplementary Files

File Name: Supplementary Movie 1
Description: A prototype stretchable liquid electret device.
